# Construction of a comprehensive fetal monitoring database for the study of perinatal hypoxic ischemic encephalopathy^✰^

**DOI:** 10.1016/j.mex.2024.102664

**Published:** 2024-03-12

**Authors:** Robert E Kearney, Yvonne W. Wu, Johann Vargas-Calixto, Michael W. Kuzniewicz, Marie-Coralie Cornet, Heather Forquer, Lawrence Gerstley, Emily Hamilton, Philip A. Warrick

**Affiliations:** a Department of Biomedical Engineering, Faculty of Medicine, McGill University, 3775 University Street, Montreal, Quebec, H3A 2B4, Canada; b Departments of Neurology and Pediatrics, University of California, San Francisco, 675 Nelson Rising Lane, Ste 411, San Francisco, CA 94158, USA; c Department of Pediatrics and Division of Research, Kaiser Permanente Northern California, 2000 Broadway, Oakland, CA 94612, USA; d Department of Pediatrics, Benioff Children's Hospital, University of California San Francisco, 550 16th St, Floor 5, San Francisco, CA 94143, USA; e Kaiser Permanente, Division of Research, 2000 Broadway, Oakland, CA 94612, USA; f PeriGen Inc.100 Regency Forest Drive, Suite 200 Cary, North Carolina 27518, USA

**Keywords:** Construction of a Comprehensive Fetal Monitoring Database for the Study of Perinatal Hypoxic Ischemic Encephalopathy, Fetal monitoring, Fetal heart rate, Uterine pressure, Labour, cardiotocography (CTG), Childbirth, Neonatal encephalopathy, Neonatal acidosis, Hypoxic-ischemic encephalopathy

## Abstract

This article describes the methods used to build a large-scale database of more than 250,000 electronic fetal monitoring (EFM) records linked to a comprehensive set of clinical information about the infant, the mother, the pregnancy, labor, and outcome. The database can be used to investigate how birth outcome is related to clinical and EFM features. The main steps involved in building the database were: (1) Acquiring the raw EFM recording and clinical records for each birth. (2) Assigning each birth to an objectively defined outcome class that included normal, acidosis, and hypoxic-ischemic encephalopathy. (3) Removing all personal health information from the EFM recordings and clinical records. (4) Preprocessing the deidentified EFM records to eliminate duplicates, reformat the signals, combine signals from different sensors, and bridge gaps to generate signals in a format that can be readily analyzed. (5) Post-processing the repaired EFM recordings to extract key features of the fetal heart rate, uterine activity, and their relations. (6) Populating a database that links the clinical information, EFM records, and EFM features to support easy querying and retrieval.

• A multi-step process is required to build a comprehensive database linking electronic temporal fetal monitoring signals to a comprehensive set of clinical information about the infant, the mother, the pregnancy, labor, and outcome.
• The current database documents more than 250,000 births including almost 4,000 acidosis and 400 HIE cases. This represents more than 80% of the births that occurred in 15 Northern California Kaiser Permanente Hospitals between 2011–2019. This is a valuable resource for studying the factors predictive of outcome.
• The signal processing code and schemas for the database are freely available. The database will not be permitted to leave Kaiser firewalls, but a process is in place to allow interested investigators to access it.

## Graphical abstract

Created with BioRender.com

**Figure:**
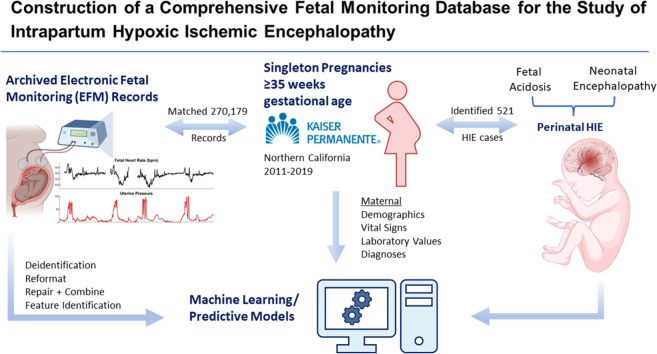

Specifications Table

**Table:** 

Subject area:	Medicine and Dentistry
More specific subject area:	Fetal monitoring and outcome prediction
Name of your method:	Construction of a Comprehensive Fetal Monitoring Database for the Study of Perinatal Hypoxic Ischemic Encephalopathy
Name and reference of original method:	*Not applicable*
Resource availability:	*M*ATLAB code can be found at: https://github.com/reklab/EM_Analysis.git https://github.com/reklab/reklab_public.git

## Method details

### Background and rationale

Neonatal hypoxic-ischemic encephalopathy (HIE) is a neurologic syndrome that results from reduced flow of oxygenated blood to the fetal or newborn brain. HIE occurs in 1–3 per 1000 term births and may cause death or neurologic disabilities such as cerebral palsy (CP). Electronic fetal monitoring (EFM) was developed in the 1970s to assess the adequacy of fetal oxygenation as a strategy to prevent HIE and is now standard of care. Yet clinical trials report that EFM usage has not reduced the rate of CP, perinatal death or HIE, but is associated with a dramatic increase in cesarean deliveries. The currently used three-category fetal heart rate (FHR) classification system, based on simple rules designed to be easy to apply at the bedside, has some utility in predicting HIE. However, Category II FHR patterns make up most tracings; these are poorly predictive of HIE and confer “indeterminate” risk. Even Category III patterns are of limited use in predicting HIE because they have low sensitivity. There is an urgent need to develop better objective methods to assess EFM that would identify more fetuses at risk of HIE in time for corrective actions. Uterine tachysystole, or excessively frequent uterine contractions, has been implicated as a preventable cause of HIE, yet studies report conflicting results. EFM research has been limited by an inability to access and manually analyze the large datasets needed to study HIE. We can now analyze digital EFM signals using automated methods to measure standard FHR patterns as well as to discover novel aspects of the tracing that may not be readily detectable by a clinician at the bedside. We hypothesize that modern signal processing and machine learning techniques can create models highly predictive of HIE by analyzing established and novel features of EFM tracings, in combination with demographic and pertinent clinical information from the mother and fetus. Our research collaboration has obtained funding from both the NIH and the Bill & Melinda Gates Foundation to investigate this hypothesis.

A major factor limiting progress in predicting the risk of HIE has been the absence of an EFM dataset comprising enough records to support machine learning efforts and a carefully curated outcome measure. Consequently, the first objective of our collaborative research was to build a large database of EFM records and link it to an extensive set of clinical information about the infant, the mother, the pregnancy, labor, and outcome. Building this dataset proved to be a challenging and complex operation. This paper will describe the methods used to build the database and provide guidance for those wishing to build their own databases.

## Background

### Overview of the database

**Fig. 1:**
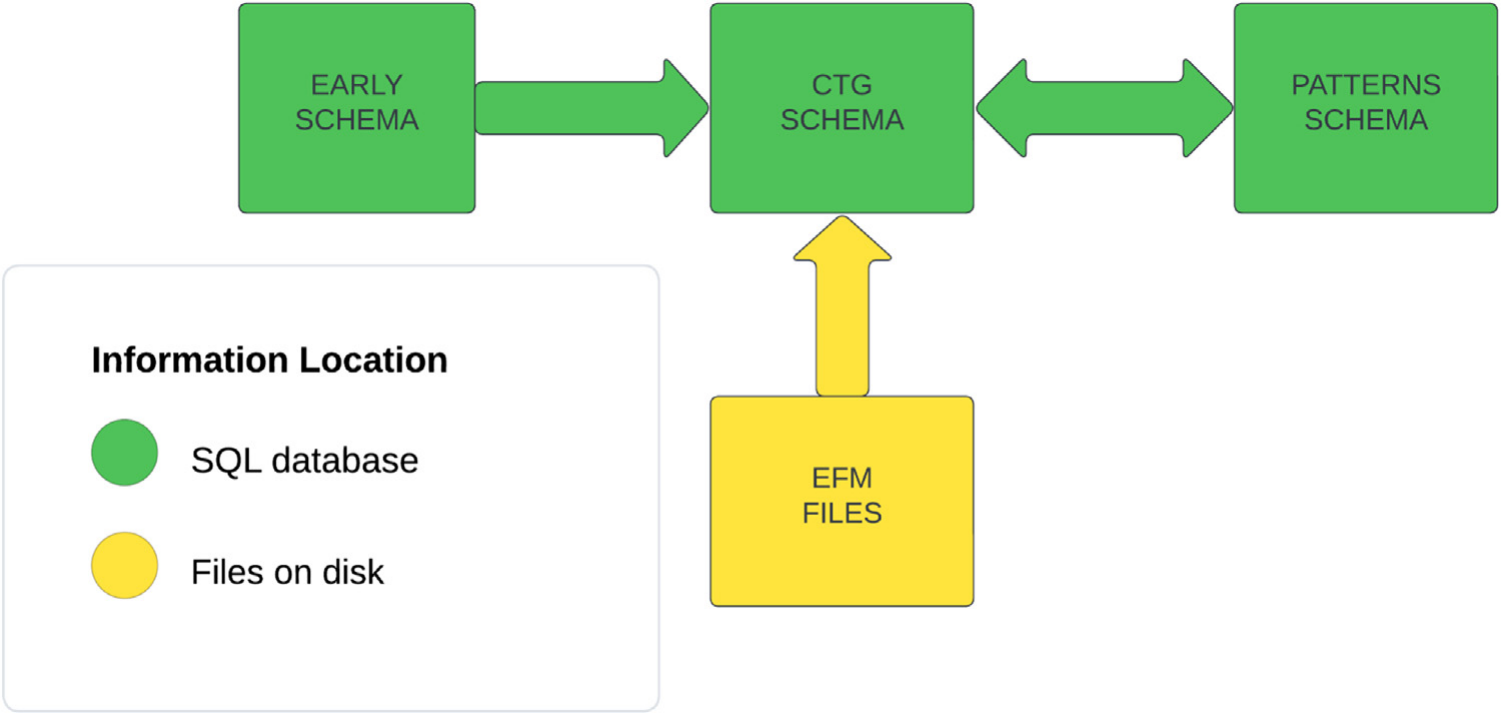
The components of the fetal monitoring database. Three schemas implemented in an SQL database: The EARLY schema holds clinical information about the mother, infant, pregnancy, and delivery. The CTG schema holds information about the processing of the EFM files, and their contents. The PATTERNS schema holds information about FHR and UA patterns. The EFM files stored on the server disk contain the raw and processed fetal heart rate (FHR), uterine activity (UA), and maternal heart rate (MHR) signals as function of time.

The overall design of our database is similar in principle to that described by Chudáček [1]. This database linked EFM signals and clinical data for a limited number of selected cases for mostly vaginal births. However, our objective was to build a database that was more comprehensive than that of Chudáček [1]. Thus, we wished to include as many records as possible from vaginal and cesarean births with both normal and pathological outcomes. Secondly, we wanted to expand the clinical information available to include clinical information about the progress of labor. Third, we wanted to include EFM records for as much of the labor as possible. Finally, we wanted to include information about important features of the EFM files. Fig. 1 illustrates the overall structure of the fetal/maternal database comprising four main components.

Three components were implemented as SQL schemas. These included:

1. The EARLY schema contains clinical information about the mother, the infant, the progress of the pregnancy and labor. The primary index of this schema is the INFANT_GUID, the infant identifier. It also contains a foreign key, TRACE_GUID, which identifies the corresponding trace file.
2. The CTG (CardioTocoGraphy) schema contains information about the location and contents of the EFM tracing files, their processing status, and some of the features computed from the EFM tracings.
3. The PATTERNS schema contains information about the processing of the EFM files to identify and quantify clinically relevant patterns in the fetal heart rate and uterine pressure signals.

The fourth component comprised the EFM trace files which were stored on the server file system.

### Overview of database construction

The construction of the database was carried out in two parallel workflows: One for the EARLY database and one for the EFM files. These two workflows are summarized in the following two sections. A detailed description of the various workflows then follows.

### EARLY

**Fig. 2:**
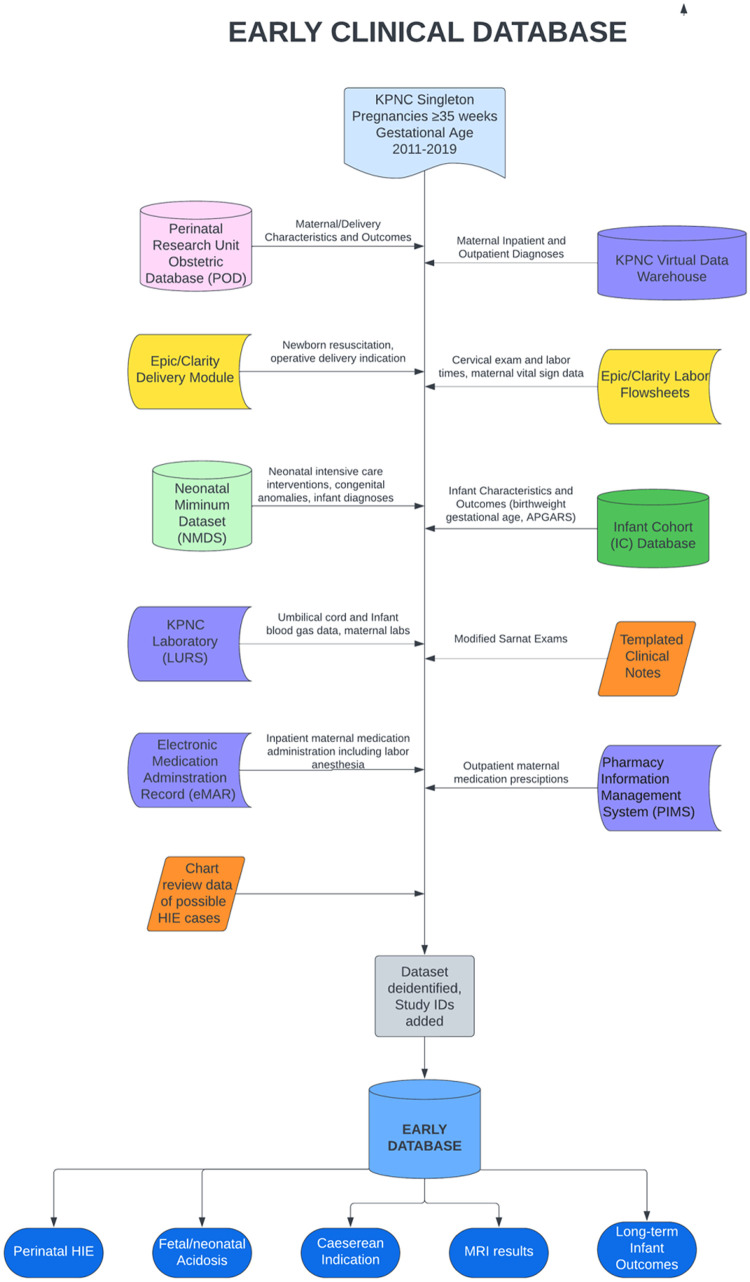
The EARLY database workflow.

The workflow for constructing the EARLY schema is illustrated in Fig. 2 comprised the construction of the EARLY database containing a wide variety of clinical data for the mother, child, pregnancy, and labor. To achieve this the Kaiser Permanente (KP) staff first identified all singleton pregnancies with a gestational age of 35 weeks or greater. They then drew data from ten different KP databases to provide a comprehensive picture of the mother, infant, pregnancy. All personal health information was removed from the database and unique, deidentified identifiers were assigned to the mother, infant, and EFM trace files. The deidentified results were entered into the EARLY schema and made available to the EARLY/MEASTRA team. Subsequently, this information was used to generate views describing the study group for each infant, the indication for cesarean section, and long-term outcome.

#### EFM workflow

**Fig. 3:**
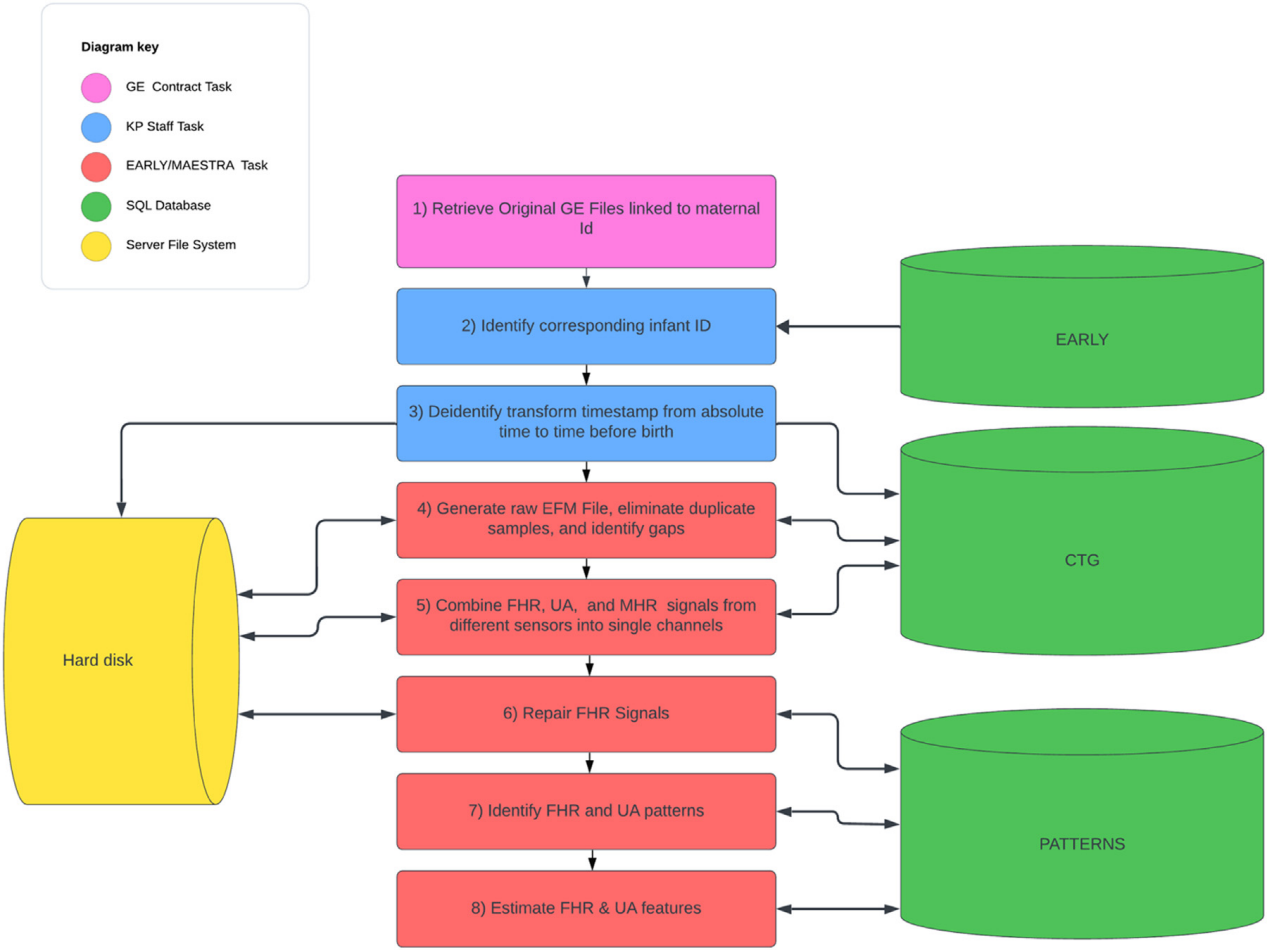
EFM workflow indicating the analyses performed by the GE, the KP staff, and EARLY MEASTRA staff. Information about the processing and contents of the EFM files was stored in the CTG and PATTERNS schemas The EFM files themselves were stored on disk.

The EFM files were acquired, preprocessed, repaired, and patterns identified using the workflow shown in Fig. 3. This workflow is similar in principle, although different in detail, from that described in [2]. The workflow proceeded as follows:

1) GE retrieved all EFM files from the various KP sites. Files were identified by the mother's MRN and recording date.
2) KP used the MRN and recording date to associate each file with a specific pregnancy and infant. KP staff removed all personal health information by assigning the file a random TRACE_GUID and transforming the timestamp from absolute time to time before birth. The deidentified file was stored in CVS form with one sample per row and transferred to the EARLY/MEASTRA team for subsequent processing.
3) The KP CVS file was processed to eliminate duplicate samples, identify gaps, and stored as a *RAW EFM* file with one channel for each unique sensor/signal combination.
4) FHR, UA, and MHR signals from different sensors were combined to generate a *Combined EFM* file with one channel for each of the three signals.
5) The *Combined EFM* file was then processed to generate a *Repaired EFM* file in which short gaps were interpolated and segments containing artifacts or excessive noise were identified as uninterpretable.
6) The *Repaired EFM* file was processed using PeriCALM Patterns software to identify clinically important patterns in the FHR (i.e. baseline, decelerations, accelerations) and UA (contractions).
7) The *Repaired EFM* files and patterns information were used to estimate a wide range of FHR and UA features for classification.

As each stage of the workflow information about the processing status, contents of the EFM file, and analysis results were stored in the CTG or PATTERNS schemas. The different EFM files were stored on the server disk for further analysis or to develop machine-learning models.

## Detailed description of the database construction

This section provides a more detailed description of the tools and methods used to generate the Fetal Monitoring database.

### Computing infrastructure

For legal and ethical reasons, KPNC imposed two restrictions on the project team:

1. No individual data would be allowed outside of the firewall of the KPNC network computing environment; all computation and analysis would have to be done within the KPNC firewall.
2. Even within the firewall, no personal health information (PHI) would be available to members of the team who were not KPNC employees.

These restrictions had important implications for how the database was built. Thus, two groups of personnel were involved in building the EFM database:

1. The KPNC Data Science Team comprises KPNC employees with full access to all data including PHI.
2. The Analysis Team comprising members of the research collaboration with access only to de-identified data.

The computing environment available behind the KPNC firewall to the Analysis Team comprised the following:

1. Each member of the Analysis Team was provided with a virtual Citrix Windows 10 workstation equipped with key software tools including Microsoft Office and Teams, MATLAB with selected toolboxes, Git Extensions for source code revision control, Python, and PyCharm for Python development. Remote access to these machines was through a two-stage KPNC security protocol.
2. All members of the Analysis Team had access to a Unix server system which acted as a file server for the Windows machines, a containerized MySQL database server, and provided NVidia GPU support for deep learning applications. The KPNC Data Science group also had access to this machine and used it to transfer deidentified EFM signals and expose database tables to the Analysis Team.

### Target population

The target population for the database included all pregnancies with an onset date in the range January 1, 2011–December 31, 2018, which led to singleton births at 15 Kaiser Permanente Northern California (KPNC) hospitals with a gestational age greater than or equal to 35 weeks, and for whom electronic fetal monitoring (EFM) data was available.

### Early schema

The EARLY schema contains clinical information describing the pre-pregnancy period, the pregnancy, and the delivery encounter. It comprises the data tables listed in Table 1. A complete data dictionary for the EARLY schema is provided as supplementary material.

**Table 1:** Tables in the EARLY schema.

Table	Description
Anesthesia	Maternal anesthesia types during the delivery encounter
Anomaly	Infant anomaly/birth defect diagnoses
Blood gas	Information about timing, nature, and values of blood gas measurements.
Cervical exam	Dilatation, effacement, and station data
Delivery	Characteristics of the delivery encounter
EDOC	Hourly summary measures of maternal features during the delivery encounter including vital sign data, lab data, labor stage
escore	Individual elements of the modified SARNAT (neurologic) exam
Infant	Infant characteristics and outcomes
Infant labs	Additional infant laboratory values including CBC, transaminases, serum creatinine, coagulation studies
Inpatient Medications	Medication, dosage, administration
Labor times	Duration, onset, second stage
Maternal	Static maternal variables
Maternal diagnoses	Diagnoses pre-pregnancy, during the pregnancy, and during the delivery encounter
Maternal diagnosis timing	For selected diagnoses, specific timing of diagnosis
NMDS	Neonatal diagnoses and procedures including stroke, seizure, seizure medications, imaging, therapeutic hypothermia
Outpatient medications	Maternal medication, dosage, dispensed
Pregnancy	Maternal characteristics unique to each pregnancy
Resuscitation	Resuscitation received in the delivery room
UDS	Results of urine drug screening during pregnancy

Data were abstracted from linked maternal and infant electronic medical records. To develop this database, we leveraged existing data resources which include the KPNC Division of Research Virtual Data Warehouse, a formal instance of the Health Care Systems Research Network's Virtual Data Warehouse [3] an independent multi-institutional standardized data model containing tables for diagnostic codes on all inpatient and outpatient encounters, inpatient electronic medication administration records, outpatient pharmacy records, and inpatient and outpatient labs; the infant cohort database which contains basic demographic, administrative, and clinical data of infant/mother dyads; the Neonatal Minimum Data Set (NMDS), a research database of curated outcomes and interventions of infants admitted to the neonatal intensive care unit [4]; and the Perinatal research unit Obstetric Database (POD), a curated obstetric research database of pregnancy diagnoses, outcomes and complications. In addition, we abstracted data directly from the electronic health record (EHR) including nursing flowsheet data (vital signs, cervical exam), neuroimaging reports, and clinical notes.

These clinical data allow us to determine a pregnancy's baseline risk for an adverse neonatal outcome based upon the maternal medical history and pregnancy. A unique feature of the dataset is the hourly record of information on maternal physiological variables (vital signs, lab data) and labor stage during the delivery encounter. These data can pair with EFM data to provide a dynamic estimation of risk as labor progresses. In addition, we will be able to evaluate the individual contribution to risk prediction of clinical data and EFM data as well as the effect of combining these two predictors.

An important function of the EARLY database was to link the tables describing the mother's and infant's clinical data with the tables for corresponding tracing files for a delivery. We used field values with globally unique identifiers (GUIDs) to provide these “crosswalk” linkages.

#### Study group definition

**Fig. 4:**
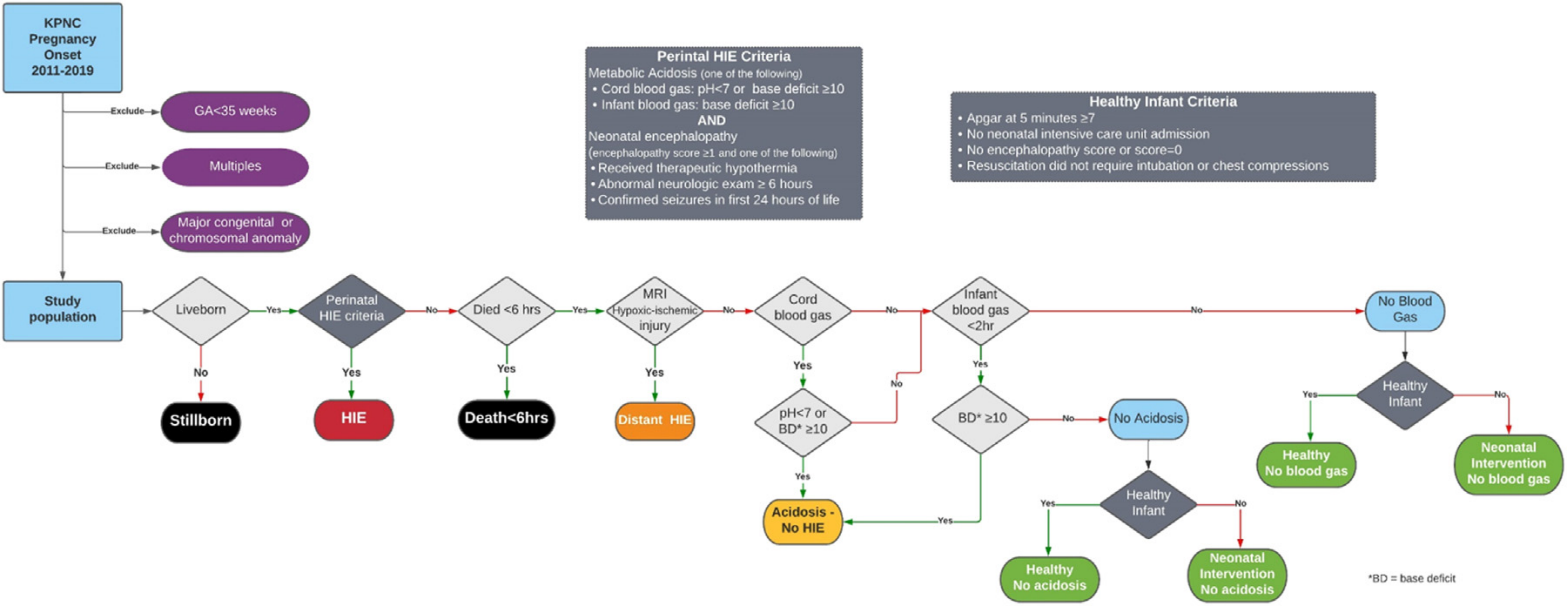
Flow chart for study group definition.

Each infant in the EARLY schema was assigned to one of eight mutually exclusive study groups based on their outcome. Fig. 4 shows the logic associated with this assignment while Table 2 provides a detailed description of or each study group.

**Table 2:** Study group definitions.

Study Group	Definition
Perinatal HIE	Acidosis on cord gas (pH <7 or BD ≥10) or infant gas (BD ≥10) AND encephalopathy score >0 AND one or more of the following: • Received therapeutic hypothermia. • Abnormal neurologic exam ≥6 hours • Confirmed seizures in first 24 hours of life
Acidosis, No HIE	Acidosis on cord gas (pH <7 or BD ≥10) or infant gas (BD ≥10) AND encephalopathy score =0 AND NONE of the following: • Received therapeutic hypothermia. • Abnormal neurologic exam ≥6 hours • Confirmed seizures in first 24 hours of life
Healthy, no cord gas or infant blood gas	No cord gas or infant blood gas AND ALL the following: • Apgar at 5 min ≥7 • Never admitted to NICU • Discharged home. • No death or hospital transfer • No encephalopathy noted. • Did not require intubation or chest compressions during newborn resuscitation. • No evidence of seizures or seizure medications prescribed. • No therapeutic hypothermia (passive, active, or transient)
Healthy, no acidosis:	ALL the following: • Cord gas pH ≥7 or BD <10; OR infant gas BD<10 • Apgar @ 5 min ≥7 • Never admitted to NICU • Discharged home. • No death or hospital transfer • No encephalopathy score/ SARNAT score recorded or encephalopathy score = 0 • Did not require intubation/chest compressions during newborn resuscitation. • No evidence of seizures or seizure medications • No therapeutic hypothermia (passive, active, or transient)
Neonatal intervention, no cord gas or infant blood gas	No cord gas or infant blood gas AND ONE OR MORE of the following: • Apgar @ 5 min <7 • Admitted to NICU • Death or hospital transfer • Encephalopathy score >0 • Required intubation/chest compressions during newborn resuscitation. • Evidence of seizures or seizure medications • Therapeutic hypothermia (passive, active, or transient)
Nenonatal Intervention, no acidosis:	Cord gas pH ≥7 BD <10; infant gas BD<!0 AND ONE OR MORE of the following: • Apgar @ 5 min <7 • Admitted to NICU • Death or hospital transfer • Encephalopathy score >0 • Required intubation/chest compressions during newborn resuscitation. • Evidence of seizures or seizure medications • Therapeutic hypothermia (passive, active, or transient)
Atypical/Distant HIE	ALL the following: • Does not meet criteria for perinatal or intrapartum HIE. • Evidence on radiographic study (Head CT or Brain MRI) of injury pattern suggestive of hypoxic/ischemic injury (e.g., injury to the deep grey matter [basal ganglia, thalamus] or watershed regions)
Death under 6 h	Infant died within 6 hours of birth

We took particular care with the assignment of infants to the HIE study group. Thus, *HIE* was defined as the presence of both neonatal acidosis and neonatal encephalopathy.

Neonatal acidosis was defined as a pH <7.0 or base deficit ≥10 mmol/L on cord blood, or a base deficit ≥10 mmol/L on the first infant gas before 2 h of age [5], [6], [7]. To identify infants with acidosis, we electronically extracted all cord and infant blood gas data from the KPNC laboratory database. We used the lowest pH of all available umbilical cord gases, whether they were arterial or venous. Similarly, we used the first infant blood gas regardless of source (arterial, capillary, or venous),

Neonatal encephalopathy was defined as an abnormal neurologic exam between 1 and 6 h of age that either persisted beyond 6 h of age; was accompanied by electrographic or focal-clonic seizures; or was treated with therapeutic hypothermia. At KPNC, clinicians are prompted in the electronic health record to perform and document a templated “encephalopathy score” or enhanced neurologic exam (ENE) for all infants with a 10-minute Apgar <7 or a cord or infant base deficit ≥10 mmol/L. This prospectively collected exam includes an assessment of all six items of the Sarnat exam [8]: level of consciousness, spontaneous activity, tone, primitive reflexes, posture, and autonomic function. Therefore, to identify all infants with neonatal encephalopathy, we reviewed the clinical newborn records of all infants with a documented abnormal ENE, as well as all infants with any of the following conditions suggestive of possible neonatal encephalopathy: acidosis; perinatal depression, i.e., 5-minute Apgar <7 and hospital stay >72 h; death within 72 h of age; discharge diagnosis of HIE or seizures; receipt of anti-epileptic medication before 72 h of age; or receipt of therapeutic hypothermia.

The severity of HIE was determined by the worst neurologic exam documented between 1 and 6 h of age. We defined moderate to severe HIE as three or more abnormalities on the ENE or seizures, and mild HIE as < 3 abnormalities on the ENE and the absence of seizures.

## EFM processing

Retrieving the electronic fetal monitoring (EFM) records and converting them into form suitable for analysis involved the complex multi-stage procedure outlined in Fig. 3. The following section provides a detailed description of each stage.

### Stage 1: Retrieval of tracing files

The EFM records from the KPNC facilities were stored on multiple General Electric (GE) Healthcare servers distributed throughout the KPNC system. GE was contracted to locate and extract EFM records from these multiple sources, including legacy systems. Extracted records (henceforth referred to as tracing files), were placed on a shared GE-KPNC server as text files in comma-separated-values (CSV) format with a unique tracing identifier. GE provided a crosswalk linking the tracing identifier to the maternal medical record numbers (MRNs). These files were formatted as repeating records with the fields shown in Table 3 GE provided the team with 19 batches of records comprising a total of 756,012 tracings.

**Table 3:** Record format of EFM files retrieved by GE at.

Field	Description
Trace Identifier	Link to the Maternal MRN
Trace DT	Time when monitor recorded the data
Recorded DT	Time when the data record was received by the server
Measurement	Name of the signal recorded
Monitor	Name of the monitor used to record the signal
Sensor	Name of the sensor used to record the signal
240 sample values	One minute of samples at 4 samples/s

### Stage 2: Linking tracing files to infants

The tracing files produced by GE had unique identifiers that were linked by a crosswalk directly to the maternal medical record numbers (MRN) which identified the mothers. However, linking the tracing files to the infants within the study group posed some difficulties.

First, the MRN field in the crosswalk was expected to contain a string of 12 digits containing the prefix ‘110’ followed by an 8-digit identifier. However, in 3% of the records, this field included nonnumeric characters; these records were not further processed. In the 99% of the remaining records, the MRNs were 7, 8, or 12 digits long. Eight digits MRNs lacked the “1100” prefix while seven-digit MRNs were missing leading zeroes. These MRNs were padded with leading zeroes as needed and the “1100” prefix added. MRNs with more than 12 digits or less than 7 were not further processed.

A second problem was that mothers could have had more than one pregnancy and delivery within the study cohort. Therefore, after linking each EFM tracing file to a specific mother, we determined which of the mother's pregnancies was associated with the tracing by matching the tracing timestamp to the infant's birthdate.

Inspection of the resulting infant/tracing linkages revealed several special cases which were handled as follows:

1. Tracing files linked to pregnancies with stillborn infants were set aside for later analysis, since these were not in the study population. They will be the topic for future analysis once methods are developed to determine the timing of the infant's demise.
2. Tracing files linked to multiple liveborn (“multiples”) were set aside and not further processed since they could not be linked to individual infants and so were excluded from the study group.
3. Some tracing files were associated with maternal MRNs linked to pregnancies spanning multiple years. Investigation revealed that these files contained concatenated data from several pregnancies during the study period. In such cases, we used the timestamps to split the tracing file into separate files for each pregnancy, and then matched them to infants according to the same criteria as previously described.
4. Occasionally multiple tracings were linked to the same pregnancy. Potential duplicates were identified by comparing the minimum and maximum timestamps in the tracings. When the minimum and maximum were identical, the individual values from the files themselves were compared. This identified 7500 pregnancies having one or more exact duplicates. In such cases, one file was retained, and the others removed from the analysis set. There were also about 600 pregnancies with more than one successfully matched non-duplicate tracing files; these were removed from analysis.
5. Tracings containing only data for more than 72 hours before birth were not analyzed. Portions of traces which occurred earlier than 72 hours before birth were not retained.

### Stage 3: Deidentification and reformatting of the GE tracing files

The KPNC Data Science Team further processed the GE tracing files to remove all personal health information (PHI). For each tracing file, the original MRN identifier was replaced with a new GUID which was linked via a crosswalk to a separate infant GUID. Furthermore, the original timestamps which included actual dates and times were replaced by the time, in milliseconds, before birth.

Then, the original wide file structure provided by GE was transformed to a tall CVS format with one record for each sample. The transformed record structure is described in Table 4. These reformatted, deidentified records were then transferred to the EARLY server where they could be accessed by the Analysis Team.

**Table 4:** Transformed tracing file record structure.

Field Name	Definition
Tracing GUID	Unique identifier for tracing
Trace DT	Time before birth when the monitor recorded the data
Recorded DT	Time before birth when the data as received by the server
Measurement	Name of the signal
Sensor	Sensor used to measure
Monitor	Monitor used to record the sensor output
Reading	Sample Value

#### Signal definition

In principle, the tracing files contained data for three measurements of interest: fetal heart rate (FHR), uterine pressure (UP), and maternal heart rate (MHR). However, in practice there were some added complexities. First, the fetal monitors had two receptacles (HR1, HR2) for FHR, one for MHR, and one for UP sensors. The two FHR receptacles could accept input from either an external ultrasound-based FHR sensor or an internal fetal scalp electrode-ECG based sensor. Thus, FHR could be found on HR1 or HR2. Furthermore, when both FHR sensors were connected simultaneously, slightly different FHR readings could be present on both HR1and HR2.

Secondly, The UP receptacle could receive information from either an external toco-based sensor or from an internal pressure sensor placed within the uterine cavity.

As a result, there were two measurement names FHR, yielding a total of four possible measurements as shown in Table 5.

**Table 5:** EFM measurement definitions.

Measurement	Definition
HR1	Fetal heart rate, in beats per minute, recorded on monitor channel #1
HR2	Fetal heart rate, in beats per minute, recorded on monitor channel #2
MHR	Maternal heart rate in beats per minute
UP	Uterine pressure uncalibrated measurement ranging from 0–100

Moreover, the measurements could be derived from the different sensors shown in Table 6

**Table 6:** Types of EFM sensors.

Measurement	Sensor	Description
HR1, HR2	External	External ultrasonic device
	FECG	Fetal ECG from internal
UP	TOCO	External sensor
	Internal	Internal pressure sensor
MHR	ECG	Maternal heart rate

Finally, there were four different types of monitors used in the study as defined in Table 7. Since different monitors could process signals differently, we felt it important to document the monitor associated with each signal.

**Table 7:** Monitor definitions.

Monitor	Description
120 Series	GE Healthcare Corometrics 120 Series fetal monitor
170 Series	GE Healthcare Corometrics 170 Series fetal monitor
Coro 250	GE Healthcare Corometrics 250 Series fetal monitor
HP 135X	Hewlett Packard 135X fetal monitor

Consequently, each signal was defined by three parameters: the measurement, sensor, and monitor.

### Stage 4: Raw EFM files

In the next stage of processing, the tracing files resulting from Stage 3 were parsed and reformatted to group the samples for each signal defined by a unique monitor/sensor/measurement combination. Two issues were uncovered during this processing:

First, there were many cases for which there were multiple samples of the same signal with the same timestamp. This likely resulted when the communication between the monitor and server was interrupted and a packet of signals had to be resent with the same timestamp. There were three distinct cases which handled as follows:

1. If none of the samples were valid, the sample was dropped.
2. If one sample was valid and the others not, the valid sample was kept.
3. If multiple samples were valid but not equal, the value from the highest row was kept since it was likely a corrected value.

**Fig. 5:**
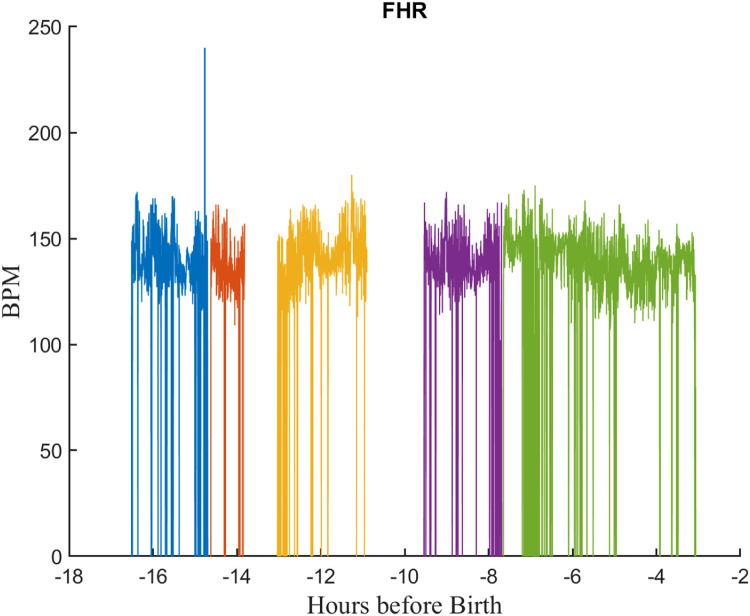
An FHR signal from a typical EFM file showing gaps and dropouts. Gaps are periods for which no samples are available. There are five contiguous segments, each shown in a different color. Dropouts are FHR samples values of zero due to noise or other monitoring problems. Each segment is shown in a different color. Note this is a compressed view showing more than 16 h of signal.

Second, in many cases, the timestamps for a signal were not continuous; there were frequent time periods (gaps) for which there were no samples. Likely causes for this included sensor disconnection from the patient and/or communication problems between the monitor and server. Fig. 5 illustrates this for a typical FHR signal. Each color corresponds to a separate segment, contiguous sets of samples separated by gaps before and after it. It should also be noted that the FHR signals also suffered from frequent dropouts–samples where the monitor was unable to estimate the heart rate because of noise or movement artifact. In such cases, the monitor stored a value of zero for the heart rate.

**Fig. 6:**
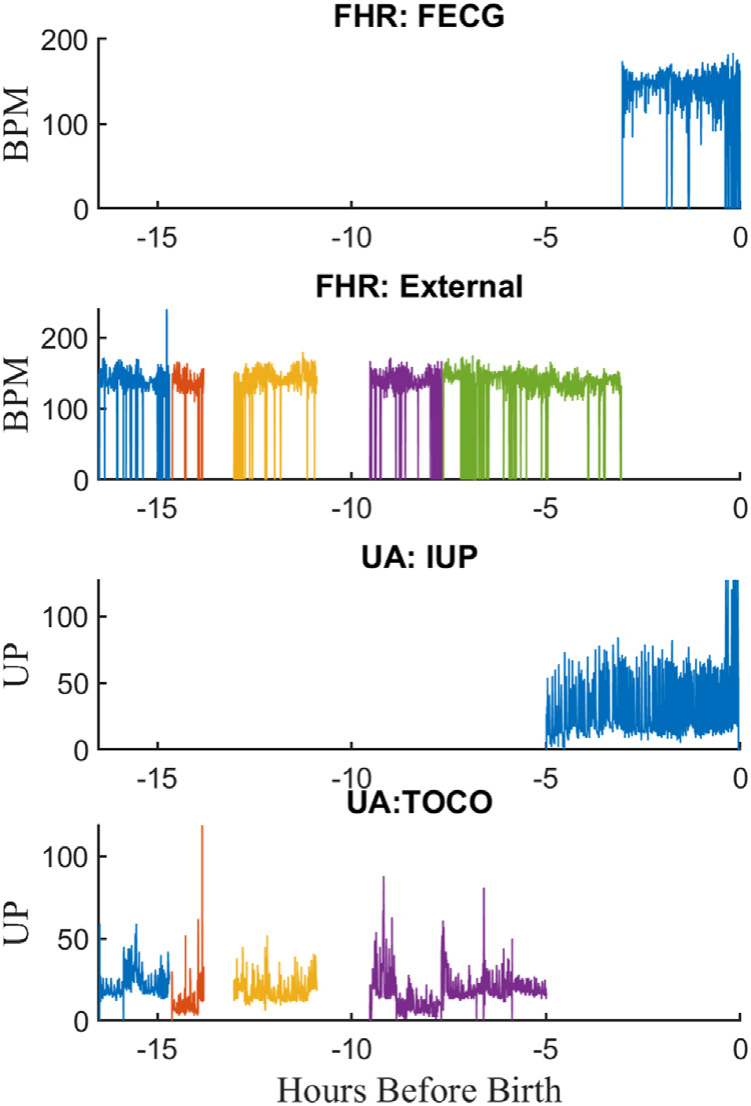
A typical “raw” EFM record showing the signals from a tracing file. Early in the record FHR is measured externally and UA with TOCO. Closer to birth the FHR is measured with FECG and UA with an internal transducer.

Thus, each signal comprised a series of segments of contiguous samples separated by gaps of varying length. To store this efficiently, we created a MATLAB class called *segdat* to store the samples and track the start and length of each segment. The *segdat* objects for all signals in a tracing file were then stored in a second custom MATLAB *efm* class. Fig. 6 shows the EFM object for a typical tracing file. The resulting data files will be referred to hence forth as “RAW EFM” files.

### Stage 5*: Combined EF*M files

We found that the RAW EFM files often contained the same measurement (e.g., FHR) from different sensors or monitors during the same labor. Thus, FHR was usually recorded via a US transducer early in the delivery but often changed to fetal ECG near the end. Similarly, uterine pressure was typically measured using a TOCO early in delivery and then replaced by an intrauterine pressure (IUP) transducer near the time of birth. Fig. 6 illustrates one such record. In addition, FHR was usually recorded using the monitors’ HR2 receptacle but in some cases both receptacles were used at different times during labor.

**Fig. 7:**
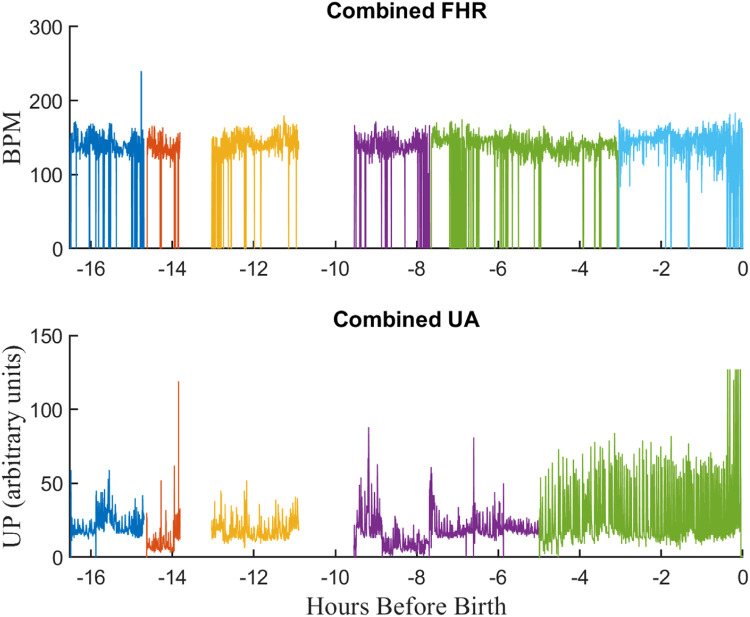
The “combined” EFM record after combining signals shown in Fig. 6.

Therefore, to provide a single signal for each measurement, the measurements for the same signal from different sensors monitors were concatenated. For times where more than one measurement was available for the same signal, the signal from the most accurate sensor (i.e., fetal ECG for FHR and IUP for UP) was given precedence. Information about the sensor used for of each signal segment was stored in the EFM file, so that the sensor used for each signal could be determined on a sample-by-sample basis if needed. The result was an “*Combined EFM* file with UP, HR, and MHR signals. Fig. 7 illustrates the *Combined EFM* file resulting from applying this procedure to the RAW EFM file of Fig. 6.

A review of the links between the *Combined EFM* files and the associated infant ID demonstrated that there were cases where *Combined EFM* files with different tracing GUIDS were linked to the same infant. Comparison of the EFM files revealed two cases.

1. In the first case, the files were identical, indicating that the original dataset provided by GE was duplicated. In such cases, one of the *Combined EFM* files was retained and the other(s) were labeled as a duplicate and removed from consideration.
2. In the second case, the *Combined EFM* files were not identical but contained overlapping versions of the signals. These records were labelled as “SPECIAL” and set aside for further analysis.

### Stage 6: FHR repair

**Fig. 8:**
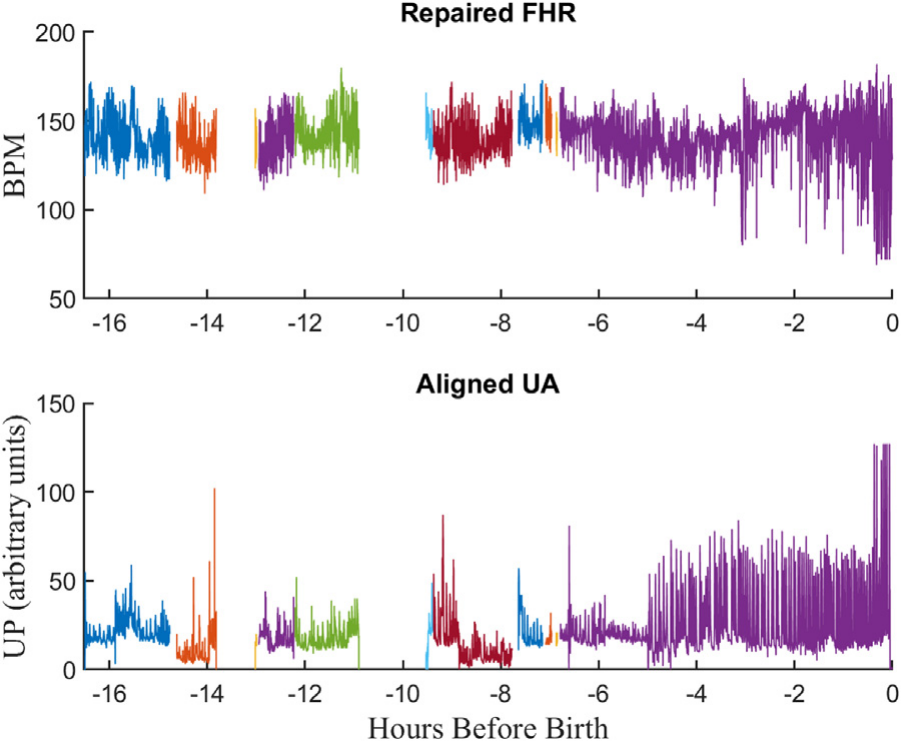
Repaired EFM files. A) Fetal heart rate B) Uterine activity. Note that the dropouts in the FHR signal have been repaired.

Finally, the FHR tracings in the *Conbined EFM* files were processed by Perigen's “PeriCalm Patterns” software to repair them [9]. This software repaired short (<15 s) FHR gaps using linear interpolation. It also recognized and removed “uninterpretable” segments. These comprised segments that were too noisy, contained interference from maternal heart rate (MHR), or suffered from long sensor dropouts. We stored the results as “repaired” EFM files which were used for subsequent signal processing and machine learning inputs. Fig. 8 illustrates the results of repairing the FHR record from the tracing file shown in Fig. 7 Note that the repair operation has corrected the frequent, short dropouts evident in Fig. 7.

## Feature detection

### Identification of clinically relevant patterns

The repaired EFM files were processed using PeriCALM Patterns, a software system from PeriGen Inc. that identified and quantified FHR and UP patterns typically considered by clinicians [10,11]. The FHR signals were first preprocessed using an ensemble of low-pass, high-pass, median and Karhunen-Loève filters. Then, PeriCALM Patterns used a long short-term memory (LSTM) network to identify FHR events and logistic regression to identify uterine contractions [10,11]. Patterns identified the events shown in Table 8.

**Table 8:** CTG events identified by patterns.

Event	Definition
FHR Baseline (BAS)	Relatively flat FHR segment with a typical range of 110–160 bpm, and a peak-to-peak variability of 5–15 bpm.
FHR Acceleration (ACC)	Segment where the FHR transiently increases more than 15 bpm for more than 15 seconds and then returns to baseline.
FHR Deceleration (DEC)	Segment where the FHR transiently decreases more than 15 bpm for more than 15 seconds and then returns to baseline.
Uninterpretable FHR (NOI)	Segment with noise or artifact PeriCALM Patterns
UP: Uterine Contraction (CON)	UP segment where the UP amplitude increases before returning to resting tone.

**Fig. 9:**
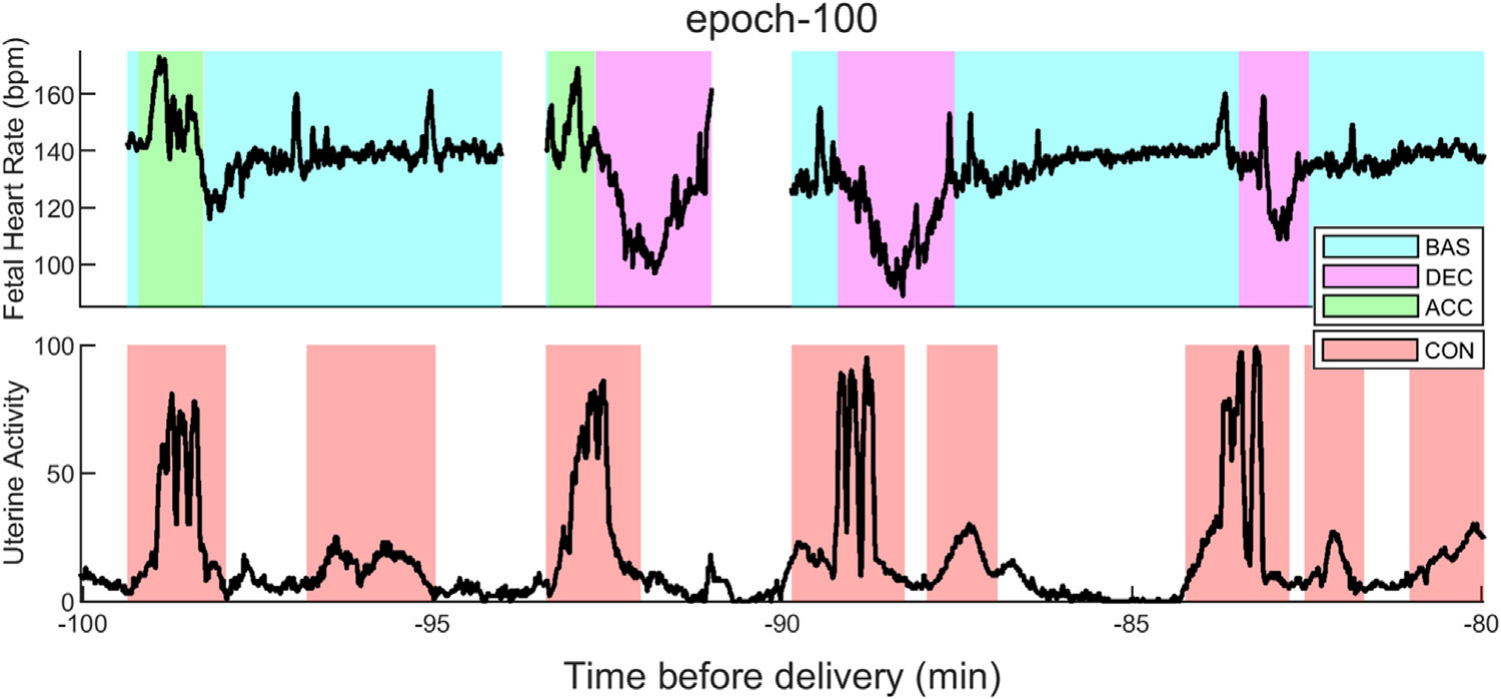
EFM patterns identified by perigen patterns: BAS – FHR baseline; DEC – FHR deceleration: FHR Acceleration; CON: Uterine Activity: contraction.

Fig. 9 shows examples of these features, identified by PeriCALM Patterns software for a typical 20-minute epoch.

Decelerations were further classified into the subtypes shown in Table 9

**Table 9:** Deceleration subtypes.

Subtype	Characteristics
Variable	Visually apparent abrupt decrease in FHR. The decrease in FHR is 15 beats per minute or greater, lasting 15 seconds or greater, and less than 2 minutes in duration. When variable decelerations are associated with uterine contractions, their onset, depth, and duration commonly vary with successive uterine contractions.
Early	Visually apparent, usually symmetrical, gradual decrease and return of the FHR associated with a uterine contraction. In most cases, the onset, nadir, and recovery of the deceleration coincide with the beginning, peak, and ending of the contraction, respectively.
Late	Visually apparent, usually symmetrical, gradual decrease and return of the FHR associated with a uterine contraction. In most cases, the onset, nadir, and recovery of the deceleration occur after the beginning, peak, and ending of the contraction, respectively.
Prolonged	Deceleration lasting at least two minutes

Each pattern detected was quantified in terms of its start and end time and the additional attributes show in Table 10.

**Table 10:** CTG pattern attributes.

Event	Attributes	Comment
UP: Contraction	Height Area	UP is uncalibrated
FHR: Baseline	Level (bpm) Variability (bpm) Slope	Level and slope computed by linear regression
FHR: Acceleration	Height (bpm) Area (beats)	
FHR: Deceleration	Height (bpm) Area (beats)	Decel subtype as described in Table 9

### Sequential pattern features

We divided the EFM signals into non-overlapping 20-minute-long epochs and discarded any samples within the epoch labeled as uninterpretable by PeriCALM Patterns within that epoch. If the remaining samples comprised less than 80% of the epoch length (less than 3840 samples), we discarded the epoch from analysis. Finally, the features listed in Table 11 were estimated from the FHR and UP signals for each of the remaining epochs. These features assess the events as marked-point processes where we quantified the sequence of events and their timing:

**Table 11:** Properties associated with a pattern.

Feature	Definition
Dwell time	The length of time that the signal spends in one event before transitioning into the next. We estimated the total length of each event type within each epoch.
Number of transitions	The number of times that an event A transitions to event B within each epoch.
Cumulative dwell time	For an event type, the sum of all previous dwell times from the beginning of clinical monitoring to the beginning of the present epoch.
Rate per 10 minutes	The number of contractions in each epoch converted to the rate per 10-minutes. This corresponds to the clinical practice where contraction rate is assessed as the number of contractions in a 10-minute period.
DEC subtype frequency	Within each epoch, the number of decelerations of each subtype expressed as their frequency.

Table 12 lists the features estimated for each event type.

**Table 12:** Features estimated for each event.

	BAS	ACC	DEC	CON	RIN
Dwell time	√▪	√▪	√▪	√▪	√▪
Cumulative dwell time			√▪	√▪	
Number of transitions to BAS		√▪	√▪		
Number of transitions to ACC	√▪		√▪		
Number of transitions to DEC	√▪	√▪			
Rate per 10 minutes				√▪	
Frequency of late DEC			√▪		
Frequency of abrupt DEC			√▪		
Frequency of others DEC			√▪		

### Fetal heart rate variability

Features describing heart rate variability were estimated from the FHR signal for each epoch. To do so, the FHR signal was first decomposed into low and high frequency components. To do so we computed the Fourier transform of the FHR and decomposed it into a low-frequency component with power from 0–30 mHz range and a high frequency component with power from 30 mHz–2 Hz. Then, the inverse Fourier transforms were computed to obtain the two components in the time domain. Finally, the two time-domain components were separated according to their corresponding event types: BAS, ACC, or DEC. Thus, the FHR was decomposed into 6 signals: three (BAS 0–30 mHz, ACC 0–30 mHz, and DEC 0–30 mHz) describing the low frequency (slow) trends of the FHR events, and three (BAS 30 mHz–2 Hz, ACC 30 mHz–2 Hz, and DEC 30 mHz–2 Hz) describing the higher frequency variability of the FHR events Table 13 summarizes the features computed for each signal.

**Table 13:** Heart rate variability features estimated.

Feature	Definition In all cases the features is the mean value for each epoch
Standard deviation	The standard deviation of the FHR values within each epoch
Area	Specific to the ACC and DEC events. We removed the average baseline from the ACC and DEC components and then estimated the area of the resulting components.
Slope	The slope obtained by performing linear regression on the event samples with time.
Height	Specific to the ACC and DEC events. We removed the average baseline from the ACC and DEC components. For the ACC height, we estimated the maximum value of the resulting component. For the DEC height, we estimated the minimum value of the resulting component.
Phase rectified signal averaging (PRSA)	PRSA is a nonparametric measure of the tendency of a signal to increase or decrease [12], We computed the acceleration capacity (AC), deceleration capacity (DC), and deceleration reserve (DR) features defined by PRSA Using a time scale parameter of T=2.5 s and an averaging window length of L=25 s.
Power spectral density (PSD)	The PSD of the events estimated using the Lomb-Scargle periodogram which is robust to gaps in signals [13]. From the estimated PSD, we focused on the powers contained in the three main bands used in fetal heart rate assessment [14]: the low-frequency (LF) band from 30–150 mHz, the movement frequency (MF) band from 150–500 mHz, and the high frequency (HF) from 0.5- 1 Hz. The LF, MF, and HF power estimates were normalized by the variance of the signal. The LF/(MF+HF) power ratio was also computed.
Approximate entropy (ApEn)	Approximate entropy is a nonlinear measure of signal complexity. Regular or periodic signals have low ApEn, while random or chaotic signals have high ApEn. ApEn(*m,r*) depends on the parameters *m*, which is the embedding dimension to compare signal regularity, and *r*, which is the similarity tolerance criterion and is given as a fraction of the standard deviation of the signal [15]. We used values of *m*=2 and values of *r*= (0.1, 0.2; 0.3)
Sample entropy (SampEn)	Sample entropy has a similar definition to the approximate entropy but does not count self-similarities in the signal, making it more robust to variations in signal length. The parameters used to compute SampEn(*m,r*) were the same than as for ApEn [16].
Hurst exponent	A measure of fractal behavior of a signal [17]. Estimated using the rescaled range analysis method [18].
Lyapunov exponent	A measure of chaos within a signal that quantifies the dependence on initial conditions [19], Estimated using an embedding dimension of 2 and delay of 1 using the Lyapunov Exponent function in MATLAB.
Correlation dimension	A measure of the fractality of the signal that has been used in previous FHR studies [20]. Estimated used an embedding dimension of 2 and a delay of 1 using the correlationDimension function in MATLAB.
Short-term variability	A measure of the variability of the signal in 2.5 s steps. It is the mean of the absolute difference of samples separated by 2.5 s in the signal [21].
Long-term irregularity	A measure of the deviations caused by slow trends in the signal [21].
Delta	The average peak-to-peak range of a signal in one-minute-long intervals It is estimated using the minimum to maximum range [21].
Interval index	The ratio of the short-term variability to the standard deviation of the samples separated by 2.5 s in the signal [21]

Table 14 summarizes the features computed for each pattern.

**Table 14:** FHR features computed by pattern.

	Component 0–30 mHz			Component 30 mHz–2 Hz		
	BAS	ACC	DEC	BAS	ACC	DEC
Mean	√▪	√▪	√▪			
Standard deviation				√▪	√▪	√▪
Slope	√▪					
Height		√▪	√▪			
Area		√▪	√▪			
PRSA AC				√▪	√▪	√▪
PRSA DC				√▪	√▪	√▪
PRSA DR				√▪	√▪	√▪
LF power				√▪	√▪	√▪
MF power				√▪	√▪	√▪
HF power				√▪	√▪	√▪
LF/(MF+HF) ratio				√▪	√▪	√▪
ApEn (2,0.1)				√▪	√▪	√▪
SampEn (2,0.1)				√▪	√▪	√▪
ApEn (2,0.2)				√▪	√▪	√▪
SampEn (2,0.2)				√▪	√▪	√▪
ApEn (2,0.3)				√▪	√▪	√▪
SampEn (2,0.3)				√▪	√▪	√▪
Hurst exponent				√▪	√▪	√▪
Lyapunov exponent				√▪	√▪	√▪
Correlation dimension				√▪	√▪	√▪
Short-term variability				√▪	√▪	√▪
Long-term irregularity	√▪	√▪	√▪			
Delta				√▪	√▪	√▪
Interval index				√▪	√▪	√▪

## EFM schemas

Two linked database schemas were used: the CTG Schema to track the processing of the EFM files, and the PATTERNS schema to hold the results of the patterns processing. Each schema is described in more detail below. Data dictionaries for each schema are provided as supplementary material.

### CTG schema

The CTG schema is used to track the pre-processing of the tracing files, store the locations of the resulting files, save statistics of the resulting EFM files, and hold information about estimated labor onset time and how labor onset was detected. Table 15 lists the tables in the CTG schema. The data dictionary for these tables is appended in the supplementary material

**Table 15:** Tables in the CTG schema.

Table Name	Functions
CTG_Status	Tracks the processing of a tracing file through its various stages including the name of the original tracing file.
signalCoverage	Coverage of each signal in a tracing file in 20 minutes epochs.
signalStats	Detailed information about each signal in a tracing file including monitor, sensor, start time and percentual coverage information about each segment.
segmentStats	Information about each segment in a tracing file.
guidStats	Summary information about each tracing file.
features4epoch	FHR and UP features are available for each epoch for each subject.
Labor_onset	Labor onset information and timing.
Labor_onset_reason	Dictionary of reasons for determining labor onset.

### Patterns schema

The patterns schema is used to track the processing of the combined files by the Patterns software, store the location of the resulting repaired files, and hold information about the FHR and UP patterns identified in each tracing file. Table 16 lists the tables in the Patterns schema and the data dictionary for these tables is provided in the supplementary material.

**Table 16:** Tables in the patterns schema.

Processing	Processing status of combined files including their location.
Dataset	Analysis dataset (study group with and without CS).
Event_Type	Identifies the type of event (BAS, ACC, DEC, CON, and NOI).
Event	Patterns event type and time extent in samples (foreign key: tracing_guid, event_type_id). For BAS attributes: FHR level and variability. ACC/DEC attributes: height and area.
Tracing	Links the patterns tracing_id with EFM tracing_guid.
Processing	For each completed major processing step, one row is generated per tracing_guid .
DomainStart	Tracing begins times with respect to the time of delivery.
Event_view	Contains all events and attributes identified from a repaired tracing file. Times expressed as time before delivery using Domainstart information.
Tracing_Epoch_EventCount	Provides number of FHR and UP events within epochs defined as non-overlapping 20-min windows.
Percentiles	For selected event metrics (e.g., DEC duration), the percentile values in increments of five percentiles.

## MATLAB support

The pre-processing and much of the subsequent analysis was pe.rformed using MATLAB and a set of novel classes designed to facilitate this work. All code is publicly available through GitHub.

## NLID_TOOLBOX

The nlid_toolbox is an object-oriented toolbox for the analysis and nonlinear identification of biological signals and system. It provides a convenient platform for the signal analysis and incorporates methods for a wide range of parametric and non-parametric methods for signal analysis and system identification [22]. The primary data class used in the nlid_toolbox is the *nldat* class designed to contain contiguous samples of one or more channels of the same size while tracking their domain values, channel names, and other valuable information.

**Fig. 10:**
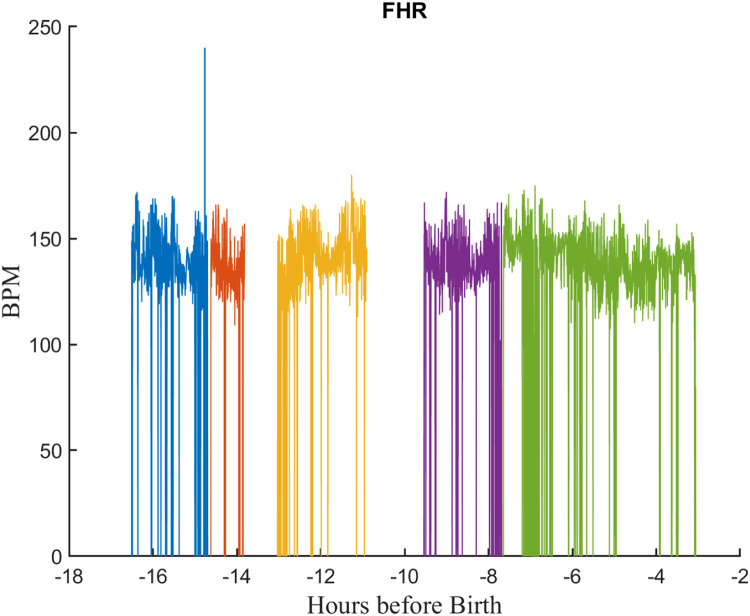
Plot of a *segdat* object for a typical FHR signal. Each segment is shown in a different color.

As mentioned previously, the EFM signals were rarely contiguous but contained frequent gaps resulting from sensor disconnects, communication problems, or noise. Thus, the EFM signals comprised a series of contiguous segments separated by gaps of different length. The *nldat* class can deal with such signals by filling the gaps with NaNs, a symbol indicating not a number. But this is a problem since it is inefficient, and the analysis methods designed for contiguous samples do not manage the NaNs correctly. To address this, the nlid_toolbox provides the *segdat* data class, a child of the *nldat* class, designed for the efficient storage and processing of such segmented signals. Thus, the *segdat* class stores signals as a continuous set of samples while keeping track of the start and length of each segment. Furthermore, the class supports a wide range of analysis methods that have been modified to work correctly with s*egdat* objects. Fig. 10 shows a plot a typical *segda*t object while Table 17 shows the properties of the corresponding *segdat* object.

**Table 17:** Properties of the *segdat* object for the FHR signal of Fig. 10.

Property	Value	Description
segInfo	{1×5 cell}	A cell array summarizing the monitor and sensor used for each segment.
parameterSet	[1×2 param]	
chanNames	{‘HR2’}	Names of the signal
chanUnits	‘BPM’	Units of the signal
domainIncr	0.25s	Sampling increment of the signal
domainName	‘Hours before Birth’	
domainStart	[-16.5108 -14.6275 -13.0372 -9.5442 -7.6442]	Start time of each segment.
domainValues	NaN	Not used
dataSet	[161,696×1 double]	Concatenated amplitude values of all signal segments.
dataSize	[193892 1]	
comment	‘0037A085D58C4CA4AB44DC923F390EBE	Comment for the signal
version	‘2.01’	Software version
parameterSet		
onsetPointer	1 26,137 37,841 68,605 95,485	Pointer to the sample number that starts each segment.
segLength	26,136 11,704 30,764 26,880 66,212	Number of samples in segment.

Table 18 summarizes some of the important methods available for *segdat* objects.

**Table 18:** Important methods for *segdat* objects.

sort	Description
cat	Concatenate *segdat* objects.
domain	Returns domains values for all segments.
filter	Filter a *segdat* object.
findGaps	Returns a list of gaps in a *segdat* object giving start and end times of each gap.
intersect	Returns the intersection of two *segdat* objects.
nl2seg	Convert a nldat object to a *segdat* using NaNs as segment separators.
nldat	Converts a *segdat* object into a nldat object filling gaps with NaNs.
segCount	Returns number of segments in a *segdat* object.
segdat	Create a *segdat* object. Converts a *nldat* object or MATLAB vector to a *segda*t object using NaNs as separators.
segGet	Returns a segment of a *segdat* object as a *nldat* object.
segPlot	Plots a *segdat* object using a distinct color for each segment.

### EFM class

**Fig. 11:**
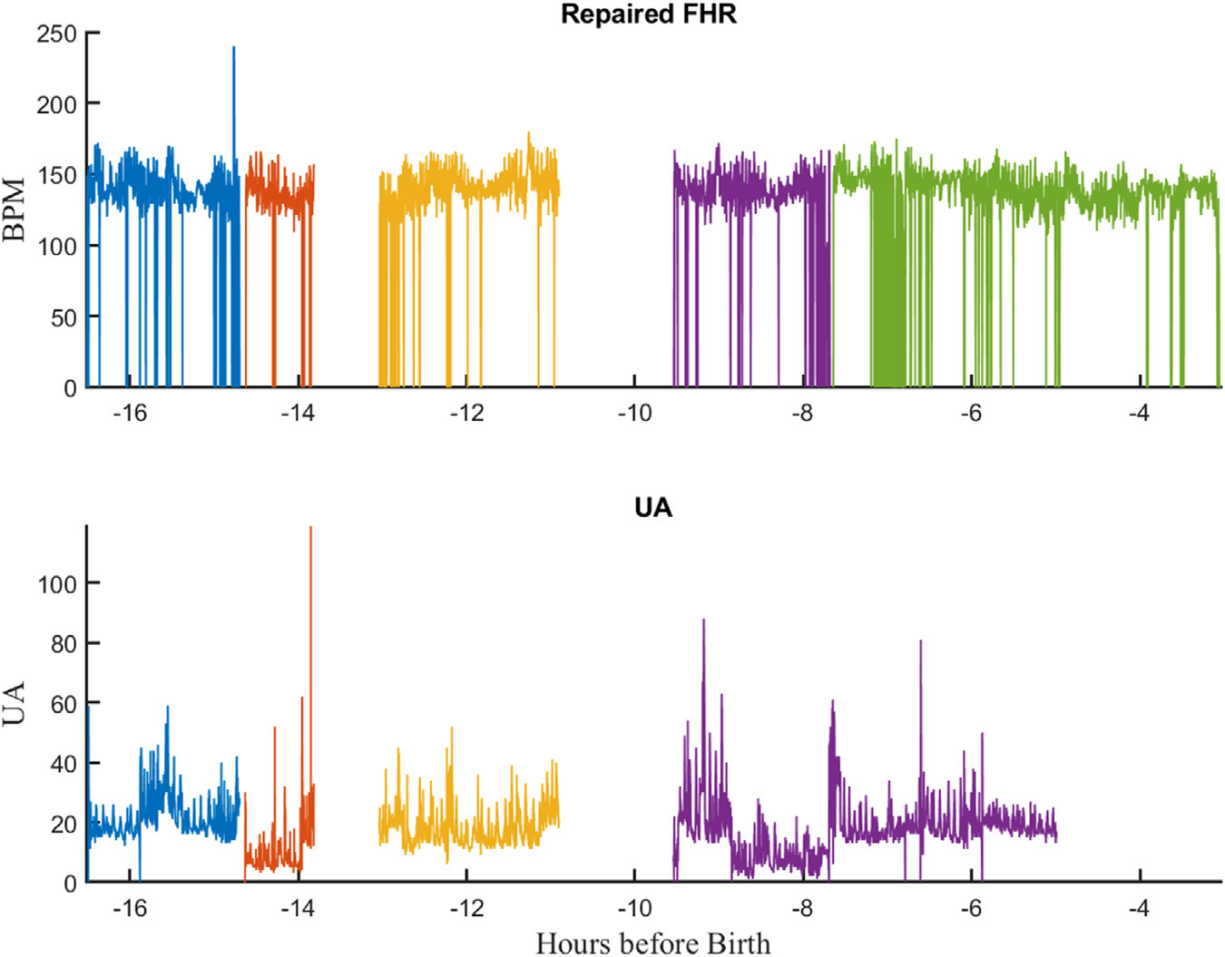
Plot of a typical EFM object. It shows the FHR and UA signal for more than 16 h before birth.

We developed the EFM class to serve as a container for the multiple *segdat* objects associated with a tracing file. Fig. 11 illustrates a typical repaired EFM record. Table 19 lists the properties of the EFM class.

**Table 19:** Properties of an EFM object.

Property	Contents	Comments
GUID	‘0037A085D58C4CA4AB44DC923F390EBE’	Tracing GUID
sourceFile	\processing\rerun\dataset14_results\ 0037A085D58C4CA4AB44DC923F390EBE.zip’	Source file from Stage 3 of EFM processing.
createDate	‘19-Jul-2023’	Date file created
signals	[1×2 struct]	Structure containing the segdat files for each signal.
comment	‘Created by emReadFile 2.01]’	Comment
version	‘2.01’	Software versions
Signal (1)	HR2	Name of first signal
measure	‘HR2’	Measure
sensor	‘external’	Sensor
monitor	‘HP 135x’	Monitor
segdat	[161,696×1×1 segdat]	Segdat object
Signal (2)	UA	Name of second signal
measure	‘UA’	Measure
sensor	‘TOCO’	Sensor
monitor	‘HP 135x’	Monitor
segdat	[134,148×1×1 segdat]	Segdat object

### *ctg* class

The *ctg* class provides a programable interface to the ctg database, tools to track the preprocessing, retrieve EFM files linked to one or more study groups. Table 20 summarizes some of the key methods for the class.

**Table 20:** Important methods of the *ctg* class.

Method	Description
combineSensors	Combine the same signal from multiple sensors into a single signal.
combinedGuid4group	Retrieves a table of EFM GUIDs and associated information after Stage3 processing (e.g.*,* combined files ready for repair). Optionally limit the selection to the study group, cesearean section, and minimum coverage.
coverage4allEpochs	Returns percentage coverage for one or more signals for all epochs as a function of trace_guid.
coverage	Determine the coverage of each signal in an EFM object as a function of epoch.
ctg	Connect to one of the schemas in the database: ctg, EARLY, or PATTERNS.
Epoch	Return data from one or more epochs in EFM object
loadFile	Load an EFM file given the file specification.
loadGUID	Load an EFM file for a specified trace_guid and process stage.
multiplot	Plot multiple EFM files in the same window.
plot	Plot a signal in an EFM file in separate panels.
readFile	Read a stage 3 CSV file, parse, convert to a EFM object.
repairedGuid4group	Retrieves table of repaired EFM GUIDs and associated information, optionally limited by studygroup and/or cesarean section status.
status4guid	Returns the pre-process status of one or more trace_guids.
traceStatus4guid	Returns status of one or more trace_guids after stage 3 processing.

## Database contents

### Processing of GE files

Table 21 summarizes the results of the first stage of processing, A total of 270,179 files were matched to infants meeting the study entrance requirements and for which there was EFM information available. The large number of files outside the study range was to be expected since GE provided all files available in the KPNC system regardless of date.

**Table 21:** Summary of KPNC file processing.

Category	N	%
Successful matched to pregnancy	270,179	35.7
Outside study range (prior)	261,664	34.6
No data within 72 hours of delivery	54,728	7.2
Empty data file	51,658	6.8
No matching maternal MRN	41,354	5.5
Outside window range (beyond)	37,811	5.0
Maternal MRN is in cohort, but pregnancy is not	16,091	2.1
Other rare/ fatal processing errors (e.g.*,* part of timestamp is missing)	9622	1.3
Associated with pregnancy of multiples	6844	0.9
Invalid MRN	5627	0.7
Pregnancy associated with stillborn infant	434	0.1
**Total**	**756,012**	**100.0**

### Babies with EFM records

Table 22 summarizes the number of records in the EARLY and CTG schemas stratified by study group. Note that:

• The “Births” column lists the number of infants in the EARLY database that meet the study requirements. It provides the upper limit on the number of EFM files that could possibly be retrieved.
• The “KPNC files” column gives the number of files linked to births in the EARLY database that were provided to the analysis team in Stage 2 of the EFM processing. The total number of files is less than the total indicated in the top row of Table 21 since 7302 of the KPNC files were found to refer to births outside of the study acceptance criteria.
• The “EFM Files” column gives the number of combined EFM files resulting from Stage 5 of the processing. These numbers were lower than the “KPNC files” due to files that were found to be duplicate, special, or to have no data.
• The “Repaired-Files” column lists the number of files after repair by PeriCalm. There is again a reduction in the numbers since some of the combined EFM had too few FHR samples to process.
• The final two rows of the table give the total number of files available at each stage and as a percentage of total births. It demonstrates that EFM files were retrieved for almost 85% of infants born during the study period. The final column of the table gives the number of repaired tracings for each group as percentage of number of births in each group. These indicated that we have EFM and clinical data for almost 80% of all births in the three study groups of most interest: ACIDOSIS-NO_HIE, HEALTHY-NO-ACIDOSIS, and HIE. This shows that all study groups were sampled equally and that there was no sampling bias.

**Table 22:** EFM file recovery.

Study Group	Births	KPNC Files	EFM Files	Repaired Files	Percent of Births in Group with Repaired Files
ACIDOSIS, NO HIE	3927	3701	3337	3223	82.1
DEATH < 6	8	8	8	8	100.0
DISTANT HIE	101	85	83	82	81.2
HEALTHY, NO ACIDOSIS	48,251	42,252	41,549	39,213	81.3
HEALTHY, NO BG	224,811	198,792	188,728	177,209	78.9
HIE	521	452	427	396	76.0
INTERVENTION, NO ACIDOSIS	9017	8070	7812	7415	82.2
INTERVENTION, NO BG	10,644	9517	9009	8549	80.3
Total	297,280	262,877	250,959	236,095	
Percent of Total Births		88.4	84.4	79.4	

Table 23 presents some baseline data regarding the patients, labor outcomes, and infants in the database.

**Table 23:** Patient and labor outcome statistics in the fetal monitoring database.

	Median	Minimum	Maximum
Maternal age (years)	31	12	56
Parity	1.0	0.0	15.0
Gravidity	2.0	0	31.0
pH*	7.24	6.47	7.71
Base*	-3.60	-33.41	27.70
Apgar 1 minute	8	0	10
Apgar 5 minute	9	0	10
Gestational age (weeks)	39	35	44
Neonate weight (g)	3400.	1300.	6090.

pH and Base values were only recorded for 45,823 AND 49,733 Infants respectively.

### EFM coverage

**Fig. 12:**
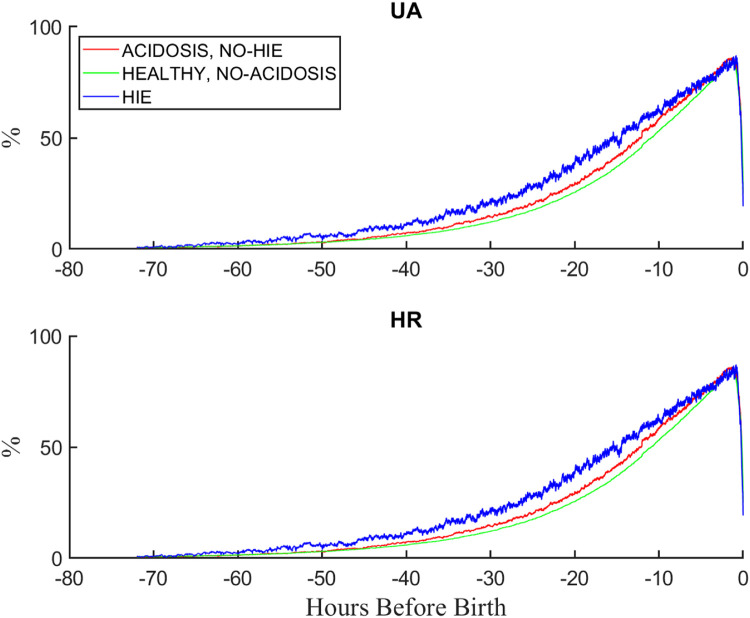
Percentage of EFM records with data as a function of time before birth.

We retrieved EFM records for up to 72 h before birth. Fig. 12 plots the percentage of records having both UP and FHR data as a function of time from 72 h before birth to recorded birth. Separate curves are shown for three main groups of interest: ACIDOSIS, NO HIE, HEALTHY, NO ACIDOSIS, and HIE. The three curves are similar, increasing gradually from low values at 72 h to a maximum just before birth and then falling dramatically. Indeed, more than 80% of all records had data within one hour of birth. Interestingly, the curve for HIE lies above that for the acidosis infants, which is in turn above the curve for the normal cases. This suggests that monitoring tended to be longer for the more severe groups.

## Limitations & directions for future work

There are some limitations to the database in its current configuration:

1. The current database contains no long-term outcome data. However, we plan to add long-term neurologic outcomes to the database in the future. The database also does not include detailed information regarding neuroimaging findings.
2. The database does not include any information regarding individual practice preferences or clinical behaviors of care providers. This is a limitation of what is available from KP.
3. Some of the FHR records may be corrupted by maternal heart rate. We currently address this issue by using the Repair process which identifies transitions from fetal to maternal heart rate and tags all episodes of maternal heart rate as artifactual. In the future, we plan to develop a more comprehensive approach to dealing with this problem using the maternal heart rate signal as a reference.
4. The database currently uses time of birth as the time reference. This is convenient but has the limitation that the time of birth is not known during labor. In future analyses, we will investigate will whether other time references, such as time at the onset of labor, might be more useful in building a HIE prediction algorithm.

## Ethics statements

The REBs of Kaiser Permanente and McGill University reviewed the study. Both REBs concluded that the study involves secondary research of identifiable private information for which consent is not required since the research involves only information collection, and the analysis of this identifiable health information is regulated by HIPAA.

## Additional Information

Publications using the Database.

As of October 2023, the database described in this paper has led to one journal publication [23], seven refereed conference papers [24], [25], [26], [27], [28], [29], [30], and eight conference abstracts [31], [32], [33], [34], [35], [36], [37].

## CRediT author statement

Robert E Kearney*: conceptualization, investigation, resources, data curation, Writing - Original Draft. writing - review and editing, funding acquisition, project administration; supervision; **Yvonne Wu:** conceptualization, investigation, resources, data curation, writing - review and editing, funding acquisition, project administration; **Johann Vargas-Calixto:** methodology, software, visualization, writing, formal analysis; **Michael Kuzniewicz:** conceptualization, investigation, resources, data curation, writing - review and editing; **Marie-Coralie Cornet:** Data Curation; Writing - review and editing; **Heather Forquer** Data Curation; **Lawrence Gerstley** Data Curation, Software; **Emily Hamilton:** Data Curation; Writing - review and editing; **Philip A. Warrick** methodology, database design, analysis and database software, visualization; writing - review and editing, supervision

## Declaration of competing interest

The authors declare that they have no known competing financial interests or personal relationships that could have appeared to influence the work reported in this paper.

## Data Availability

We expect that the results of this pilot study will spawn additional future investigations that may require further model building and/or merging with other datasets. Thus, other research groups may submit proposals to further study the EFM records in the database. Although the EFM data will not be permitted to leave Kaiser firewalls, the EARLY and EDOC datasets residing within the Division of Research will be made available to interested investigators through the following process:(1) Interested parties will submit a proposal to the Kaiser Division of Research that includes Kaiser researcher Dr. Michael Kuzniewicz (or his designate) as a Co-Investigator. (2) The proposal will be reviewed and approved by the Kaiser Permanente Northern California Institutional Review Board for the Protection of Human Subjects. (3) A Data Use Agreement between the interested parties and the Division of Research will be executed.(4) A Data Use Agreement between the interested parties and the Division of Research will be executed.

## Appendix Supplementary materials

Image, application 1

Image, application 2

Image, application 3

Image, application 4

Image, application 5

## References

[1] Chudacek V. (2014). Open access intrapartum CTG database. BMC Pregnancy ChildBirth.

[2] Mendis, L., et al., Computerised cardiotocography analysis for the automated detection of fetal compromise during labour: a review. Bioengineering, 2023. 10(9). 10.3390/bioengineering10091007 PMC10525263 37760109

[3] VDW data model, https://hcsrn.org/resources/vdw/

[4] Escobar G.J. (1997). Rapid retrieval of neonatal outcomes data: the Kaiser Permanente neonatal minimum data set. Qual. Manage Health Care.

[5] Azzopardi D.V. (2009). Moderate hypothermia to treat perinatal asphyxial encephalopathy. N. Engl. J. Med..

[6] Shankaran S. (2005). Whole-body hypothermia for neonates with hypoxic-ischemic encephalopathy. N. Engl. J. Med..

[7] Wu Y.W. (2022). Trial of erythropoietin for hypoxic-ischemic encephalopathy in newborns. N. Engl. J. Med..

[8] Power B.D. (2019). The modified sarnat score in the assessment of neonatal encephalopathy: a quality improvement initiative. Ir. Med. J..

[9] https://customer.perigen.com/peritrain-pericalm-patterns/

[10] Warrick P., Hamilton E., Macieszczak M. (2005). 2005 IEEE International Joint Conference on Neural Networksdoi.

[11] Warrick P.A., E H. (2016). 2016 Computing in Cardiology Conference (CinC).

[12] Rivolta M.W. (2020). Theoretical value of deceleration capacity points to deceleration reserve of fetal heart rate. IEEE Trans. Biomed. Eng..

[13] Clifford G.D., Tarassenko L. (2005). Quantifying errors in spectral estimates of HRV due to beat replacement and resampling. IEEE Trans. Biomed. Eng..

[14] Signorini M.G. (2003). Linear and nonlinear parameters for the analysis of fetal heart rate signal from cardiotocographic recordings. IEEE Trans. Biomed. Eng..

[15] Pincus S.M., Viscarello R.R. (1992). Approximate entropy: a regularity measure for fetal heart rate analysis. Obstet. Gynecol..

[16] Richman J.S., Moorman J.R. (2000). Physiological time-series analysis using approximate entropy and sample entropy. Am. J. Physiol. Heart. Circ. Physiol..

[17] Abry P. (2013). Proceedings of the 26th IEEE International Symposium on Computer-Based Medical Systems.

[18] Mandelbrot B.B., Wallis J.R. (1969). Robustness of the rescaled range R/S in the measurement of noncyclic long run statistical dependence. Water Resour. Res..

[19] Rajendra Acharya U. (2006). Heart rate variability: a review. Med. Biol. Eng. Comput..

[20] Ribeiro M. (2021). Non-linear methods predominant in fetal heart rate analysis: a systematic review. Front. Med..

[21] Gonçalves H., Ayres-de-Campos D., Bernardes J., Reissland N., Kisilevsky B.S. (2016). Fetal Development: Research on Brain and Behavior, Environmental Influences, and Emerging Technologies.

[22] Westwick D., Kearney R.E. (2004). Annual International Conference of the IEEE Engineering in Medicine and Biology Society.

[23] Cornet M. (2023). Perinatal hypoxic-ischemic encephalopathy: incidence over time within a modern US birth cohort. Peds. Neuro..

[24] Vargas-Calixto J. (2022). Annual International Conference of the IEEE Engineering in Medicine & Biology Society.

[25] Vargas-Calixto J. (2023). Computing in Cardiology.

[26] Vargas-Calixto J. (2023). Annual International Conference of the IEEE Engineering in Medicine and Biology Society.

[27] Vargas-Calixto J. (2022). Computing in Cardiology.

[28] Vargas-Calixto J. (2023). IEEE-EMBS International Conference on Biomedical and Health Informatics (BHI’23).

[29] Degbedzui D.K. (2022). Computing in Cardiology.

[30] Degbedzui D.K. (2023).

[31] Vargas-Calixto J. (2023). The 7th Biological and Biomedical Engineerig Symposium.

[32] Degbedzui D.K. (2023). Applying scattering transfrom and deep learning to electoric fetal monitoring signals in The 7th Biological and BioMedical Engineering Symposium.

[33] Cornet M.-C. (2023). Pediatric Academic Societies Conference.

[34] Cornet M.C. (2022). Pediatric Academic Society Conference.

[35] Cornet M.C. (2022). Pediatric Academic Society Conference.

[36] Cornet M. (2021). Child Neurology Society.

[37] Vargas-Calixto C.A.J. (2021). The Second International Summer School On Technologies and Signal Processing in Perinatal Medicine (TSPPM).

