# Supplementary material for: Construction of a comprehensive fetal monitoring database for the study of perinatal hypoxic ischemic encephalopathy
Source: MethodsX. 2024 Mar 12;12:102664. doi: 10.1016/j.mex.2024.102664 (PMC10957432; doi:10.1016/j.mex.2024.102664)
Supplement: Supplementary file 1 [file mmc1.docx]

| **Variable** | **Type** | **Len** | **Format** | **Informat** | **Label** | **Definition** |
| --- | --- | --- | --- | --- | --- | --- |
| MRN | Char | 10 | $10. | $10. | MRN | Maternal MRN |
| ENC_ID | Num | 8 |  |  | ENC_ID | Delivery encounter’s Encounter ID from Clarity |
| HET | Num | 8 | DATETIME20. | DATETIME20. | HET | Delivery encounter admission timestamp |
| DATE_HOUR | Num | 8 | DATETIME20. | DATETIME20. | DATE_HOUR | Timestamp for hourly data |
| LAPS2_CLASSIC | Num | 8 |  |  | LAPS2_CLASSIC | LAPS2 classic severity score at the hour |
| LAPS2_OB24 | Num | 8 |  |  | LAPS2_OB24 | LAPS2_OB24 score at the hour |
| LAPS2_PP02 | Num | 8 |  |  | LAPS2_PP02 | LAPS2_PP02 score at the hour |
| C03AP | Num | 8 | 2. | 2. | C03AP | 0/1 indicator for whether there was an AP combined outcome within 3 hours of that hour |
| C06AP | Num | 8 | 2. | 2. | C06AP | 0/1 indicator for whether there was an AP combined outcome within 6 hours of that hour |
| C12AP | Num | 8 | 2. | 2. | C12AP | 0/1 indicator for whether there was an AP combined outcome within 12 hours of that hour |
| C03PP | Num | 8 | 2. | 2. | C03PP | 0/1 indicator for whether there was a PP combined outcome within 3 hours of that hour |
| C06PP | Num | 8 | 2. | 2. | C06PP | 0/1 indicator for whether there was a PP combined outcome within 6 hours of that hour |
| C12PP | Num | 8 | 2. | 2. | C12PP | 0/1 indicator for whether there was a PP combined outcome within 12 hours of that hour |
| CAPTHSHR | Num | 8 | 2. | 2. | CAPTHSHR | 0 = no antepartum adverse outcome occurred during this hour  1 = the antepartum adverse outcome occurred at this hour |
| CPPTHSHR | Num | 8 | 2. | 2. | CPPTHSHR | 0 = no postpartum adverse outcome occurred during *this* hour  1 = the postpartum adverse outcome occurred at this hour |
| EAPTIME | Num | 8 |  |  | EAPTIME | Elapsed time in the AP phase at this hour |
| EPPTIME | Num | 8 |  |  | EPPTIME | Elapsed time in the PP phase at this hour |
| ETSROM | Num | 8 |  |  | ETSROM | Time in hours since ROM |
| ETSFEVER | Num | 8 |  |  | ETSFEVER | Time in hours since first occurrence of fever (temp >= 100.4) |
| ETWFEVER | Num | 8 |  |  | ETWFEVER | Time in hours that the subject actually had fever (temp >=100.4) |
| ETSLGE75 | Num | 8 |  |  | ETSLGE75 | Time in hours since the first instance of a LAPS2 24 ≥ 75 |
| ETWLGE75 | Num | 8 |  |  | ETWLGE75 | Time in hours that the subject actually had LAPS2 24 ≥ 75 |
| ETATDIL4 | Num | 8 |  |  | ETATDIL4 | Time in hours since cervical dilation was ≥ 4 cm  IF {this hour is the first hour where dilation is ≥ 4} set to = 0  IF {all dilation measurements prior to this hour are < 4} set to = 0  IF {all dilation measurements prior to this hour are missing} set to missing  ELSE = this hour minus 1st hour where dilation was ≥ 4 cm |
| ETATDIL6 | Num | 8 |  |  | ETATDIL6 | Time in hours since cervical dilation was ≥ 4 cm  IF {this hour is the first hour where dilation is ≥ 4} set to = 0  IF {all dilation measurements prior to this hour are < 4} set to = 0  IF {all dilation measurements prior to this hour are missing} set to missing  ELSE = this hour minus 1st hour where dilation was ≥ 4 cm |
| ETATDIL8 | Num | 8 |  |  | ETATDIL8 | Time in hours since cervical dilation was ≥ 4 cm  IF {this hour is the first hour where dilation is ≥ 4} set to = 0  IF {all dilation measurements prior to this hour are < 4} set to = 0  IF {all dilation measurements prior to this hour are missing} set to missing  ELSE = this hour minus 1st hour where dilation was ≥ 4 cm |
| ROM | Num | 8 | 2. | 2. | ROM | Rupture of membranes Status:  0 = Rupture of membranes did not occur in this hour  1 = Rupture of membranes did occur in this hour  2 = Rupture of membranes occurred prior to this hour |
| DELIV | Num | 8 | 2. | 2. | DELIV | 0 = Delivery did not occur in this hour  1 = Delivery occur in this hour |
| OUTCOME | Num | 8 | 2. | 2. | OUTCOME | 0= Any one or more of the EDOC adverse outcomes did not occur this hour  1= Any one of the EDOC adverse outcomes did occur at this hour |
| RBCTRNSF | Num | 8 | 2. | 2. | RBCTRNSF | 0 = the first unit of blood was not given at this hour  1 = the first unit of blood was given at this hour |
| PRELAPS | Num | 8 |  |  | PRELAPS | PreLaps – use value from latest OB24 LAPS2 score |
| PML | Num | 8 |  |  | PML | Poor Man’s Lactate (PML) – use latest value (latest ANIONGAP/latest BICARB) |
| ANIONGAP | Num | 8 |  |  | ANIONGAP | Latest value for anion gap |
| TEMP | Num | 8 |  |  | TEMP | Highest temp in the hour |
| HRTRT | Num | 8 |  |  | HRTRT | Highest HR in the hour |
| RESP | Num | 8 |  |  | RESP | Highest resp rate in the hour |
| BPSYS | Num | 8 |  |  | BPSYS | Lowest systolic BP in the hour |
| BPDIA | Num | 8 |  |  | BPDIA | Lowest diastolic BP in the hour |
| SHOCK | Num | 8 |  |  | SHOCK | Shock index: highest HR divided by lowest systolic BP, within the hour |
| O2SAT | Num | 8 |  |  | O2SAT | Lowest pulse oximetry reading in the hour |
| NEURO | Num | 8 |  |  | NEURO | Latest neuro status from nursing flowsheets. 1= normal, 2=ambiguous, 3=abnormal, 4=extremely abnormal. |
| PH | Num | 8 |  |  | PH | The latest value for arterial pH |
| LACT | Num | 8 |  |  | LACT | The latest value for lactate |
| SODIUM | Num | 8 |  |  | SODIUM | The latest value for sodium |
| BILI | Num | 8 |  |  | BILI | The latest value for bilirubin |
| BICARB | Num | 8 |  |  | BICARB | The latest value for bicarbonate |
| BUN | Num | 8 |  |  | BUN | The latest value for BUN |
| CREAT | Num | 8 |  |  | CREAT | The latest value for creatinine |
| BUNCREAT | Num | 8 |  |  | BUNCREAT | The latest value for BUN/CREATININE |
| ALBUMIN | Num | 8 |  |  | ALBUMIN | The latest value for albumin |
| GLUCOSE | Num | 8 |  |  | GLUCOSE | The latest value for glucose |
| HEMAT | Num | 8 |  |  | HEMAT | The latest value for hematocrit |
| WBC | Num | 8 |  |  | WBC | The latest value for WBC |
| PACO2 | Num | 8 |  |  | PACO2 | The latest value for PaCO2 |
| PAO2 | Num | 8 |  |  | PAO2 | The latest value for PaO2 |
| TROP | Num | 8 |  |  | TROP | The latest value for troponin I |
| MAG | Num | 8 |  |  | MAG | The latest value for serum magnesium |
| AST | Num | 8 |  |  | AST | The latest value for AST |
| ALT | Num | 8 |  |  | ALT | The latest value for ALT |
| LDH | Num | 8 |  |  | LDH | The latest value for LDH |
| URIC | Num | 8 |  |  | URIC | The latest value for uric acid |
| PLT_CT | Num | 8 |  |  | PLT_CT | The latest value for platelet count |
| STATION | Char | 10 | $10. | $10. | STATION | The latest value for fetal station. Can be missing. Range from -5 to +5 including 0 |
| DILATION | Num | 8 |  |  | DILATION | The latest value for cervical dilation. Can be missing. RTBange 0-10 |
| M_STATION | Num | 8 | 2. | 2. | M_STATION | Missingness indicator.  0= Station was recorded during the hour  1= Station was not recorded during the hour |
| M_DILATION | Num | 8 | 2. | 2. | M_DILATION | Missingness indicator.  0= Dilation was recorded during the hour  1= Dilation was not recorded during the hour |
| M_MAG | Num | 8 | 2. | 2. | M_MAG | Missingness indicator.  0= Magnesium was recorded during the hour  1= Magnesium was not recorded during the hour |
| M_AST | Num | 8 | 2. | 2. | M_AST | Missingness indicator.  0= AST was recorded during the hour  1= AST was not recorded during the hour |
| M_ALT | Num | 8 | 2. | 2. | M_ALT | Missingness indicator.  0= ALT was recorded during the hour  1= ALT was not recorded during the hour |
| M_LDH | Num | 8 | 2. | 2. | M_LDH | Missingness indicator.  0= LDH was recorded during the hour  1= LDH was not recorded during the hour |
| M_URIC | Num | 8 | 2. | 2. | M_URIC | Missingness indicator.  0= Uric acid was recorded during the hour  1= Uric acid was not recorded during the hour |
| M_PLT_CT | Num | 8 | 2. | 2. | M_PLT_CT | Missingness indicator.  0= Platelet count was recorded during the hour  1= Platelet count was not recorded during the hour |
| M_PRELAPS | Num | 8 | 2. | 2. | M_PRELAPS | Missingness indicator.  0= Prelaps score was recorded during the hour  1= Prelaps score was not recorded during the hour |
| M_PML | Num | 8 | 2. | 2. | M_PML | Missingness indicator.  0= Poor Man’s Lactate (PML) was recorded during the hour  1= Poor Man’s Lactate (PML) was not recorded during the hour |
| M_ANIONGAP | Num | 8 | 2. | 2. | M_ANIONGAP | Missingness indicator.  0= Anion Gap was recorded during the hour  1= Anion Gap was not recorded during the hour |
| M_TEMP | Num | 8 | 2. | 2. | M_TEMP | Missingness indicator.  0= Temp was recorded during the hour  1= Temp was not recorded during the hour |
| M_HRTRT | Num | 8 | 2. | 2. | M_HRTRT | Missingness indicator.  0= HR was recorded during the hour  1= HR was not recorded during the hour |
| M_RESP | Num | 8 | 2. | 2. | M_RESP | Missingness indicator.  0= RESP was recorded during the hour  1= RESP was not recorded during the hour |
| M_BPSYS | Num | 8 | 2. | 2. | M_BPSYS | Missingness indicator.  0= BPSYS was recorded during the hour  1= BPSYS was not recorded during the hour |
| M_BPDIA | Num | 8 | 2. | 2. | M_BPDIA | Missingness indicator.  0= BPDIA was recorded during the hour  1= BPDIA was not recorded during the hour |
| M_SHOCK | Num | 8 | 2. | 2. | M_SHOCK | Missingness indicator.  0= SHOCK was recorded during the hour  1= SHOCK was not recorded during the hour |
| M_O2SAT | Num | 8 | 2. | 2. | M_O2SAT | Missingness indicator.  0= O2SAT was recorded during the hour  1= O2SAT was not recorded during the hour |
| M_NEURO | Num | 8 | 2. | 2. | M_NEURO | Missingness indicator.  0= NEURO was recorded during the hour  1= NEURO was not recorded during the hour |
| M_PH | Num | 8 | 2. | 2. | M_PH | Missingness indicator.  0= PH was recorded during the hour  1= PH was not recorded during the hour |
| M_LACT | Num | 8 | 2. | 2. | M_LACT | Missingness indicator.  0= LACT was recorded during the hour  1= LACT was not recorded during the hour |
| M_SODIUM | Num | 8 | 2. | 2. | M_SODIUM | Missingness indicator.  0= SODIUM was recorded during the hour  1= SODIUM was not recorded during the hour |
| M_BILI | Num | 8 | 2. | 2. | M_BILI | Missingness indicator.  0= BILI was recorded during the hour  1= BILI was not recorded during the hour |
| M_BICARB | Num | 8 | 2. | 2. | M_BICARB | Missingness indicator.  0= BICARB was recorded during the hour  1= BICARB was not recorded during the hour |
| M_BUN | Num | 8 | 2. | 2. | M_BUN | Missingness indicator.  0= BUN was recorded during the hour  1= BUN was not recorded during the hour |
| M_CREAT | Num | 8 | 2. | 2. | M_CREAT | Missingness indicator.  0= CREAT was recorded during the hour  1= CREAT was not recorded during the hour |
| M_BUNCREAT | Num | 8 | 2. | 2. | M_BUNCREAT | Missingness indicator.  0= BUNCREAT was recorded during the hour  1= BUNCREAT was not recorded during the hour |
| M_ALBUMIN | Num | 8 | 2. | 2. | M_ALBUMIN | Missingness indicator.  0= ALBUMIN was recorded during the hour  1= ALBUMIN was not recorded during the hour |
| M_GLUCOSE | Num | 8 | 2. | 2. | M_GLUCOSE | Missingness indicator.  0= GLUCOSE was recorded during the hour  1= GLUCOSE was not recorded during the hour |
| M_HEMAT | Num | 8 | 2. | 2. | M_HEMAT | Missingness indicator.  0= HEMAT was recorded during the hour  1= HEMAT was not recorded during the hour |
| M_WBC | Num | 8 | 2. | 2. | M_WBC | Missingness indicator.  0= WBC was recorded during the hour  1= WBC was not recorded during the hour |
| M_PACO2 | Num | 8 | 2. | 2. | M_PACO2 | Missingness indicator.  0= PACO2 was recorded during the hour  1= PACO2 was not recorded during the hour |
| M_PAO2 | Num | 8 | 2. | 2. | M_PAO2 | Missingness indicator.  0= PAO2 was recorded during the hour  1= PAO2 was not recorded during the hour |
| M_TROP | Num | 8 | 2. | 2. | M_TROP | Missingness indicator.  0= TROP was recorded during the hour  1= TROP was not recorded during the hour |
