# Supplementary material for: Construction of a comprehensive fetal monitoring database for the study of perinatal hypoxic ischemic encephalopathy
Source: MethodsX. 2024 Mar 12;12:102664. doi: 10.1016/j.mex.2024.102664 (PMC10957432; doi:10.1016/j.mex.2024.102664)

Working copy Maestra Early ERD

Michael W. Kuzniewicz | October 27, 2023

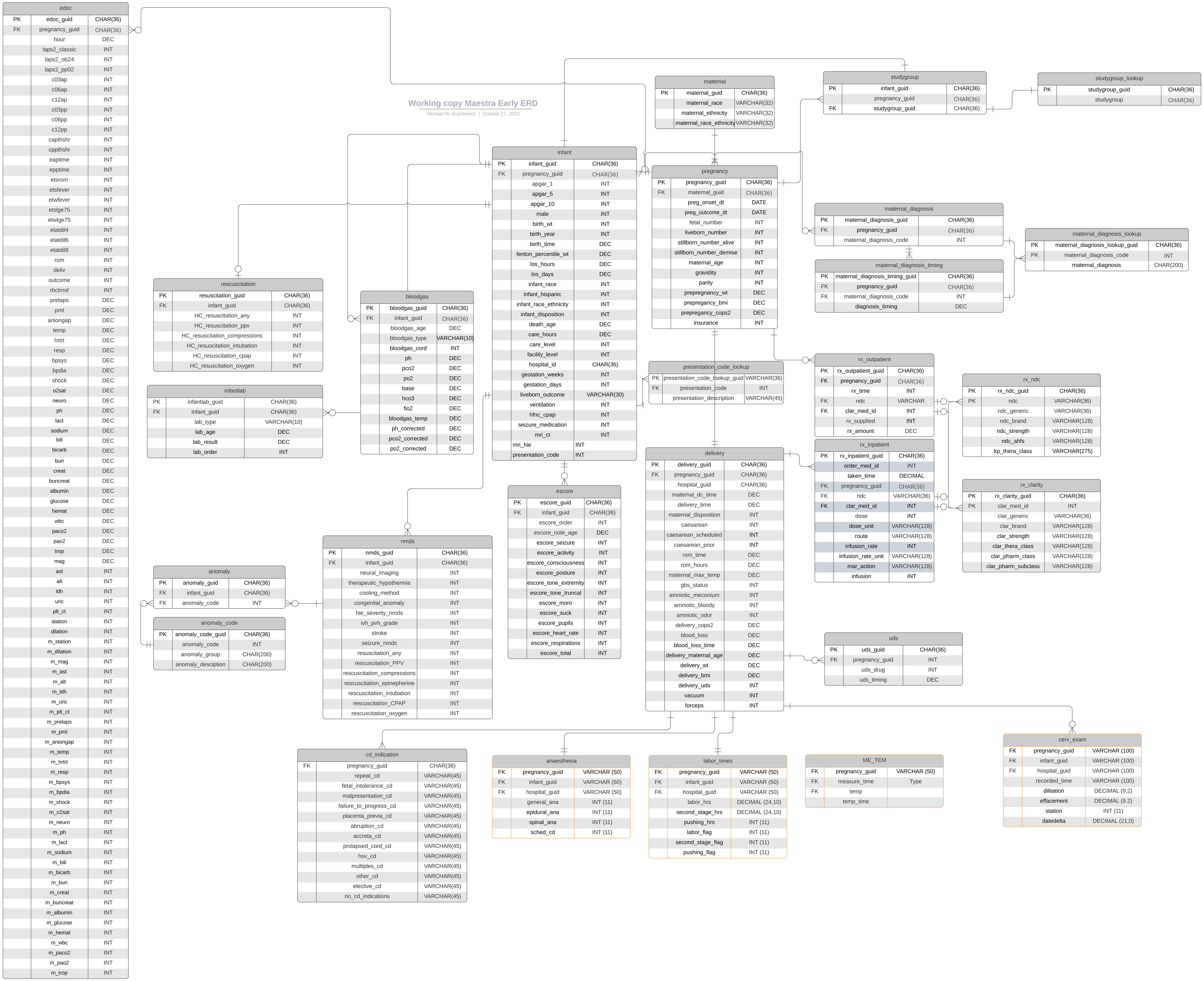

Supplement: Supplementary file 2 [file mmc2.pdf]
